# Ecological Interactions of Cyanobacteria and Heterotrophs Enhances the Robustness of Cyanobacterial Consortium for Carbon Sequestration

**DOI:** 10.3389/fmicb.2022.780346

**Published:** 2022-02-11

**Authors:** Maryam Ataeian, Yihua Liu, Angela Kouris, Alyse K. Hawley, Marc Strous

**Affiliations:** ^1^Department of Geoscience, University of Calgary, Calgary, AB, Canada; ^2^Department Microbiome Science, Max Planck Institute for Developmental Biology, Tübingen, Germany; ^3^School of Engineering, University of British Columbia Okanagan, Kelowna, BC, Canada

**Keywords:** cyanobacterial consortium, soda lakes, ecological niche, metagenomics, protein/SIP

## Abstract

Lack of robustness is a major barrier to foster a sustainable cyanobacterial biotechnology. Use of cyanobacterial consortium increases biodiversity, which provides functional redundancy and prevents invading species from disrupting the production ecosystem. Here we characterized a cyanobacterial consortium enriched from microbial mats of alkaline soda lakes in BC, Canada, at high pH and alkalinity. This consortium has been grown in open laboratory culture for 4 years without crashes. Using shotgun metagenomic sequencing, 29 heterotrophic metagenome-assembled-genomes (MAGs) were retrieved and were assigned to *Bacteroidota, Alphaproteobacteria*, *Gammaproteobacteria*, *Verrucomicrobiota*, *Patescibacteria*, *Planctomycetota*, and Archaea. In combination with metaproteomics, the overall stability of the consortium was determined under different cultivation conditions. Genome information from each heterotrophic population was investigated for six ecological niches created by cyanobacterial metabolism and one niche for phototrophy. Genome-resolved metaproteomics with stable isotope probing using ^13^C-bicarbonate (protein/SIP) showed tight coupling of carbon transfer from cyanobacteria to the heterotrophic populations, specially *Wenzhouxiangella.* The community structure was compared to a previously described consortium of a closely related cyanobacteria, which indicated that the results may be generalized. Productivity losses associated with heterotrophic metabolism were relatively small compared to other losses during photosynthesis.

## Introduction

Ecological interactions among phototrophs and heterotrophs are at the core of all light-exposed ecosystems and the entry into much of the biological carbon cycling on Earth (Paerl and Pinckney, 1996; Falkowski et al., 1998; Grossart et al., 2006; Azam and Malfatti, 2007). The diversity and complexity inherent to many natural ecosystems presents challenges in defining the ecological niches that describe the complex web of these interactions. The assembly of microbial communities and understanding the metabolic roles of each population is an important step to engineering microbial communities to carry out a desired function. Cultivation of a simplified model microbial community, collected from a complex natural ecosystem offers an opportunity to study these metabolic interactions (Beliaev et al., 2014; Aharonovich and Sher, 2016; Biller et al., 2016). For many phototrophic communities, cyanobacteria are the primary producers, capturing solar energy and providing fixed carbon and molecular oxygen to heterotrophic populations. Simplified cyanobacterial consortia, obtained from a complex natural community by selective enrichment, can provide insight into the community assembly processes for productive natural environments (Paerl and Pinckney, 1996; Paerl et al., 2000).

The high growth rate of many cyanobacteria makes them excellent candidates for biotechnology. However, there remain many challenges with the implementation of cyanobacterial technologies at scale. One of these is robustness (Quinn et al., 2012). Cultures that work well in the laboratory may crash in large scale, non-sterile production systems with more variable environmental conditions. This lack of robustness is a major barrier to achieving sustainable and economically feasible biotechnology (Shurin et al., 2013). One potential solution to increase stability and robustness of cyanobacterial biotechnology is to use the benefits of biodiversity by using a designed or naturally occurring cyanobacterial consortium instead of a monoculture (Cole et al., 2014; Duffy et al., 2017; Sharp et al., 2017).

Two fundamental ecological concepts contribute to robustness of microbial consortia (Brockhurst et al., 2007). First, the diverse members of the consortium occupy the various available ecological niches. By filling up the available niches, less resources will be available to invading species and it becomes more difficult for invading organisms to successfully colonize and potentially destabilize the production ecosystem (Moran and Miller, 2007; Jousset et al., 2011; Tan et al., 2015; Gallien and Carboni, 2017). Second, biodiversity among the organisms occupying a niche improves the overall stability of the process by creating functional redundancy (Louca et al., 2018). When variability in environmental conditions or viral attack knocks out one organism, others can take over its function.

In nature, highly productive cyanobacterial mats benefit from mutualistic phototroph–heterotroph interactions that are based on nutrient cycling (Christie-Oleza et al., 2017). These interactions occur in a context of recurring ecological niches, as shown by specific and conserved diurnal gene expression patterns of marine heterotrophs (Aylward et al., 2015). Phototrophs produce large amounts of dissolved organic matter (DOM) which is the main source of carbon and energy for the heterotrophs (McCarren et al., 2010). Some of this organic matter is excreted as fermentation products such as formate, acetate, lactate, ethanol, and H_2_ at night (Stal and Moezelaar, 1997). Low molecular weight carbohydrates and free amino acids can be released as osmolytes following osmotic shock (Reed et al., 1986; Hagemann, 2011). Organic matter can also be released in the form of outer membrane lipid vesicles containing proteins, DNA, and RNA (Biller et al., 2014). Finally, organic matter can be released by lysis of cyanobacterial cells, for example, due to viral attack by cyanophages (Puxty et al., 2018). Some *Bacteroidetes* have been shown to feed on remains of dead cyanobacterial cells (Ben Hania et al., 2017). Some *Planctomycetes* and *Verrucomicrobia* encode glycoside hydrolases and sulfatases in their genomes (Orellana et al., 2021). These enzymes become highly expressed in response to availability of sulfated polysaccharides, suggesting the ability of these species to use sulfated glycopolymers, part of the cyanobacterial cell wall, as a carbon source (De Philippis et al., 1998; Wegner et al., 2013; Spring et al., 2016).

In return, heterotrophs may benefit phototrophs by remineralizing DOM. DOM mostly consists of large molecules called biopolymers. Most microorganisms, including cyanobacteria, are unable to import and process these biopolymers. Heterotrophic bacteria remineralize biopolymers with extracellular enzymes, recycling essential elements contained in these molecules to primary producers. For example, phototrophs benefit from released and recycled nitrogen, phosphorus, and metals (Azam, 1998; Pedler et al., 2014; Christie-Oleza et al., 2015, 2017). Some heterotrophs can also harvest solar energy not used by cyanobacteria as supplementary energy source using Bacteriochlorophyll (Croce and Van Amerongen, 2014) or Proteorhodopsins (Gómez-Consarnau et al., 2007). Heterotrophs also protect phototrophs against opportunistic predators using a range of target-specific inhibitors such as antibacterial (James et al., 1996), anti-larval (Holmström et al., 1992), antialgal (Egan et al., 2001), or antifungal (Franks et al., 2006) molecules. Some *Gammaproteobacteria* facilitate iron uptake by producing siderophores that bind iron and increase its solubility (Amin et al., 2009; D’Onofrio et al., 2010). Exogenous sources of essential vitamins such as cobalamin (vitamin B12), thiamine (vitamin B1), and biotin (vitamin B7) have been shown to be essential to many marine microalgal species (Croft et al., 2005, 2006; Sañudo-Wilhelmy et al., 2012). Mathematical models have proposed various strategies for delivery of these vitamins, including symbiotic interactions (Thompson et al., 2012; Grant et al., 2014) or passive delivery through the marine microbial loop where nutrients and vitamins from lysed cells in deep water move to the photic zone by upwelling (Karl, 2002). *Sulfitobacter* species promote diatom cell division by providing growth factors such as indole-3-acetic acid (Amin et al., 2015). Some *Sulfitobacter*, *Colwellia*, and *Pibocella* species have been shown to reduce oxidative stress by producing catalase, superoxide dismutase and glutathione reductase in the vicinity of *diatoms* and *Prochlorococcus* bacteria (Hünken et al., 2008; Morris et al., 2008).

Since in biotechnology, cyanobacteria are generally provided with excess nutrients and vitamins, ecological interactions improving nutrient availability are less relevant in that context. While cultivating cyanobacteria in a community may improve robustness, it also potentially decreases biomass yield and productivity because of the conversion of cyanobacterial biomass into heterotrophic biomass. For aerobic heterotrophs, for every carbon atom they assimilate, they respire up to one additional carbon atom as a source of energy (Roller and Schmidt, 2015). Therefore, the overall productivity may be reduced due to heterotrophic catabolism. On the other hand, it is unclear whether cyanobacterial pure cultures are able to convert all the produced carbon into cellular biomass. Overflow of fermentation products (Cano et al., 2018), release of osmolytes (Hagemann, 2011), and remains of dead cells (Aguilera et al., 2021) are examples of organic carbon produced by cyanobacteria that may remain unused. These unused resources provide opportunities for successful invasion of cultivation systems, ultimately leading to a culture crash (Shelef and Soeder, 1980; Forehead and O’Kelly, 2013).

We previously enriched a cyanobacterial consortium from microbial mats of four alkaline soda lakes in BC, Canada, at high pH and alkalinity (Sharp et al., 2017). These soda lakes harbor diverse communities of microorganisms, supporting the growth of high cell density phototrophic mats (Brady et al., 2013). Recently, a core microbiome of <100 shared bacterial lineages was described for Canadian and Asian soda lake systems (Vavourakis et al., 2018; Zorz et al., 2019). Although each lake has a different microbial community structure, a nearly identical cyanobacterial consortium was enriched from each lake in laboratory photobioreactors (PBRs) (Sharp et al., 2017). More than 80% of the consortium consisted of a *Candidatus* Phormidium alkaliphilum (Ataeian et al., 2021) maintaining a high and robust biomass productivity of 15.2 ± 1.0 g/m^2^/day (Ataeian et al., 2019) during 4 years of crash-free growth in open laboratory culture.

Here we investigated the niche partitioning within the *Ca.* P. alkaliphilum consortium and adaptation to different PBRs with different pH and nitrogen sources (ammonium, urea, and nitrate) using genomics and proteomics. Based on the literature reviewed above, we postulated, *a priori*, six ecological niches for the consortium’s heterotrophs and assigned each heterotroph to one of these niches based on its observed gene content. The niches were: (1) phototrophy, use of (2) fermentation products, (3) compatible solutes, (4) storage compounds, (5) polysaccharides of the cyanobacterial cell wall, and (6) predation of cyanobacteria. Stable isotope probing with ^13^C-labeled bicarbonate, followed by proteomics (protein/SIP) was used to determine the flow of carbon in the consortium. We compared the community structure to a previously described bacterial consortium containing a closely related cyanobacterium. Potential biomass productivity losses associated with heterotrophic catabolism were estimated based on metagenomes and proteomes.

## Materials and Methods

### Cultivation of Cyanobacterial Consortium and Sample Preparation

From microbial mats growing in alkaline soda lakes located in BC, Canada (Sharp et al., 2017), a cyanobacterial consortium was enriched in a set of planar PBRs using medium “low-pH ammonium” (see below and Ataeian et al., 2019).

After 1 year of enrichment, the culture was transferred to tubular PBRs and was grown as biofilms on a mesh (Supplementary Figure 14A). Four sets of tubular PBRs were maintained, each with a different medium (“high-pH nitrate,” “high-pH urea,” “low-pH nitrate” and “low-pH ammonium,” see below) to compare biomass growth rates, as previously described (Ataeian et al., 2019). Briefly, 4 L of growth medium was circulated through each 2 L tubular PBRs at a flow rate of 10 mL/min. Daily measurements were performed for pH, soluble nitrogen species, bicarbonate, and carbonate concentrations. Depletion of soluble nitrogen in the medium after four to 5 days of cultivation was used to determine the harvesting time for the biomass. Once the growth cycle was completed, the accumulated biomass was manually detached and washed off the mesh, until no visible biomass remained. The next growth cycle was inoculated with 10% of the harvested biomass. Part of the harvested biomass was immediately frozen at -80°C for genomic and proteomic analyses.

For all four experiments, the growth medium contained: K_2_HPO_4_ (1.44 mM), MgSO_4_⋅7H_2_O (1 mM), CaCl_2_⋅2H_2_O (0.17 mM), KCl (6 mM), NaCl (0.43 mM), ferric ammonium citrate (10 mg/L), and 1 mL/L of trace metal solution containing: Titriplex III (EDTA) (500 mg), FeSO_4_.7H_2_O (200 mg), ZnSO_4_.7H_2_O (10 mg), MnCl_2_.4H_2_O (3 mg), H_3_BO_3_ (30 mg), CoCl_2_.6H_2_O (20 mg), CuCl_2_.2H_2_O (1 mg), NiCl_2_.6H_2_O (2 mg), Na_2_MoO_4_.2H_2_O (3 mg) per 1,000 mL of solution. Cultures were grown in duplicates under four different pH and nitrogen source conditions: low-pH ammonium (4 mM NH_4_Cl, initial pH 8.3), low-pH nitrate (1 mM NH_4_Cl, 3 mM NaNO_3_, initial pH 8.3), high-pH nitrate (1 mM NH_4_Cl, 3 mM NaNO_3_, initial pH 10.4), high-pH urea (1 mM NH_4_Cl, 3 mM Urea, initial pH 10.4). Sodium bicarbonate and sodium carbonate concentrations were mixed to adjust the initial pH of the medium (low-pH: 460 mM NaHCO_3_ and 20 mM Na_2_CO_3_, high-pH: 35 mM NaHCO_3_ and 230 mM Na_2_CO_3_). All experiments where performed at ∼25°C. Light was provided with full spectrum LED lights (Model T5H0; 6400K, Sunblaster Holdings ULC, Langley, BC, Canada) using a 16 h: 8 h light to dark cycle.

Following the 2 years of cultivation in tubular PBRs, biomass grown in “high-pH nitrate” medium showed the highest growth rate and was transferred to a set of stirred 10 L glass bottle PBRs with “high-pH nitrate” medium (Supplementary Figure 14B). These PBRs were stirred at 300–330 RPM as previously described (Ataeian et al., 2021). In these systems, the biomass grew as small biomass aggregates that rapidly co-aggulated during harvesting, after the stirrer was stopped. The final DNA sample used was taken 1 year after the transfer to the stirred PBR.

### Shotgun Metagenome Sequencing and Data Processing

For the tubular bioreactors, genomic DNA was extracted from the harvested biomass using FastDNA SPIN Kit for Soil protocol (MP Biomedicals, Santa Ana, CA, United States). The protocol provided by the manufacturer was used with minor modifications; the centrifugation time was raised to 10 min and 5.5 M guanidine thiocyanate was used for additional purification steps. DNA concentrations were measured using a Qubit 2.0 fluorometer (Thermo Fisher Scientific, Canada). Metagenomic library preparation and DNA sequencing was conducted at the Center for Health Genomics and Informatics in the Cumming School of Medicine, University of Calgary. DNA was sequenced paired end 2 × 150 bp with a 300 cycle mid-output reagent cartridge on the Illumina NextSeq 500 sequencer. The samples were sheared to approximately 350 bp *via* Covaris sonication, and the libraries were prepared with NEB Ultra II library preparation kit. After adaptor ligation, the average library sizes ranged from 471–483 bp. The adaptors were the NEBNext Multiplex Oligos for Illumina Set 1 and Set 2. Raw reads were passed through an in-house quality control program to cut primers and adapters as well as filter out artifacts and low-quality reads as previously described (Saidi-Mehrabad et al., 2013; Kleiner et al., 2017). After read quality control, 45,308,504, 42,813,618, 43,709,454, and 41,705,594 reads remained for the low-pH ammonium, low-pH nitrate, high-pH nitrate, and high-pH urea datasets, respectively. BBmerge was used with default setting to merge the overlapping reads. Reads from all four samples were combined and assembled into contigs using MEGAHIT v1.0.3 (Li et al., 2016). Contigs of <500 bp were discarded. Sequencing coverage was estimated by mapping reads from quality controlled fastq files to assembled contigs using BBMap. Contigs were binned into Metagenome-Assembled-Genomes (MAGs) using MetaBAT version 2.12.1 with following options “-a depth.txt -saveTNF saved_2500.tnf -saveDistance saved_2500.dist -v -superspecific -B 20 –keep” (Kang et al., 2015). MAGs were further assessed for contamination and completeness using CheckM version 1.0.8 (Parks et al., 2015). GTDBtK was used for classification of the MAGs (Parks et al., 2018). MetaErg was used for annotation of the MAGs (Dong and Strous, 2019). fastANI (Jain et al., 2018) was used for comparison of MAGs across samples to MAGs previously obtained from Kulunda soda lakes (Vavourakis et al., 2018) and Cariboo Plateau soda lakes (Zorz et al., 2019). 16S rRNA gene sequences were obtained with hyloFlash2^1^ and were associated with MAGs based on phylogeny and sequencing coverage. Genes for production of secondary metabolites were identified with antiSMASH (Blin et al., 2019).

Genomic DNA was also extracted from stirred PBRs. DNA was extracted from pelleted biomass using a modified version of the FastDNA SPIN Kit for Soil protocol (MP Biomedicals) as previously described (Costa et al., 2009), with minor modifications. The samples were processed in a bead beater twice for 30 s at setting 4.5 m/s. To prepare the genomic DNA sequencing library, the Nextera DNA Flex Library Prep protocol (Illumina) was used following the manufacturer’s protocol. Shotgun metagenomic sequencing (2 × 300 bp) was performed using an Illumina MiSeq sequencer. Read quality control and assembly was performed as described in Ataeian et al. (2021).

### Phylogenetic Analysis

**FIGURE 1:**
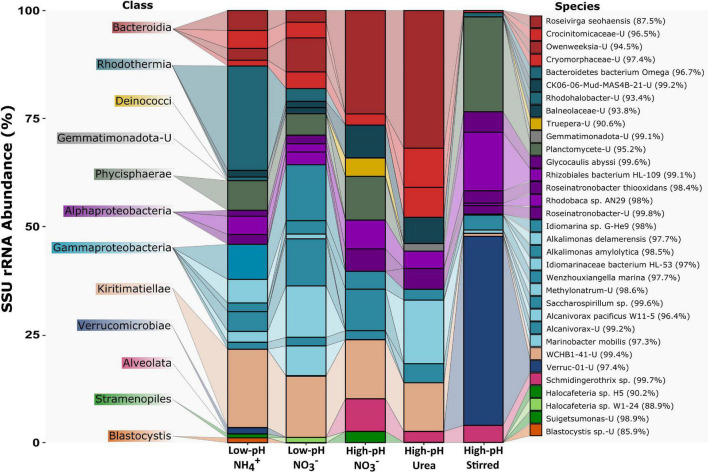
Community structure of the heterotrophs in the *Candidatus* “Phormidium alkaliphilum” consortium based on SSU rRNA extracted from metagenomes. The figure shows the abundance and taxonomic classification of populations as determined by phyloFlash. The classification is shown at class (left) and species (right) level. The percentage identity to the reference species is shown next to each species. *Candidatus* “Phormidium alkaliphilum,” >72% abundant in all experiments, is not shown. Despite its name, Bacteroidetes bacterium Omega, is affiliated with Rhodothermia. Data in Supplementary Table 1.

The GTDB tool kit with function classify_wf was used to construct all the phylogenetic trees (Parks et al., 2018). All 91 MAGs recently obtained from the original Cariboo mats (Zorz et al., 2019) and 871 MAGs from sediments of the Kulunda Steppe (Vavourakis et al., 2018) as well as all 18 MAGs from *Phormidium* OSCR consortium were included as reference sequences. gtdbtk classify_wf was used for taxonomic classification of genomes. After pplacer process all the provided genomes and finds the maximum-likelihood placement of each genome in the Genome Taxonomy DataBase Toolkit (GTDB-Tk) reference tree, the individual trees were reconstructed. phyloFlash_compare.pl was used for clustering in Figure 1. Samples were clustered by their similarity in terms of taxonomic content.

### Protein Extraction, Peptide Preparation, and Metaproteomics

Protein was extracted from biomass from tubular PBRs. Four technical replicates were used for each condition. SDT-lysis buffer (0.1 M DTT) was added to lysing matrix bead tubes A (MP Biomedicals) containing PBR biomass in a 10:1 ratio. Tubes were bead-beated in an OMNI Bead Ruptor 24 for 45 s at 6 ms^–1^. Then tubes were incubated at 95°C for 10 min, spun down for 5 min at 21,000 × *g* and tryptic peptides were isolated from pellets by filter-aided sample preparation (FASP) (Wisniewski et al., 2009) with modification from Hamann et al. (2016). One-dimensional LC-MS/MS using a block-randomized design (Oberg and Vitek, 2009) was performed. For each run, 1200 ng of extracted peptides were loaded onto a 5 mm, 300 μm ID C18 Acclaim^®^ PepMap100 pre-column (Thermo Fisher Scientific) using an UltiMate™ 3000 RSLCnano Liquid Chromatograph (Thermo Fisher Scientific) and desalted on the pre-column. Peptides were separated on a 50 cm × 75 μm analytical EASY-Spray column packed with PepMap RSLC C_18_, 2 μm material (Thermo Fisher Scientific). The analytical column was connected directly to the Q Exactive Plus hybrid quadrupole-Orbitrap mass spectrometer (Thermo Fisher Scientific) *via* an Easy-Spray source. Peptides were separated on the analytical column at a flow rate of 225 nL/min using a 260 min gradient and mass spectra acquired in the Orbitrap (Petersen et al., 2016).

Expressed proteins were identified and quantified with Proteome Discoverer version 2.0.0.802 (Thermo Fisher Scientific), using the Sequest HT node as described previously (Petersen et al., 2016). The protein-level false discovery rate (FDR) was restricted to below 5% (FidoCT *q*-value < 0.05) (Serang et al., 2010) for high confidence identifications (FidoCT *q*-value < 0.01) and medium confidence identifications (FidoCT *q*-value 0.01–0.05).

Relative protein abundances were estimated based on normalized spectral abundance factors (NSAFs) method (Florens et al., 2006). Abundances of MAGs in the metaproteome were estimated by dividing the sum of the relative abundances for all of its expressed proteins by the sum of the relative protein abundances for all expressed proteins. The identification database was created using predicted protein sequences of binned and unbinned contigs, after filtering out highly similar proteins (>95% amino acid identity) with cd-hit (Li and Godzik, 2006). The cRAP protein sequence database^2^ of common laboratory contaminating proteins were added to the final database. In total 11,426 proteins were identified. After removing the proteins identified as cyanobacterial, the rest was used. The number of proteins was reduced by only including proteins that had at least four NSAF values greater than 0 in all replicates of one condition. The mass spectrometry proteomics data have been deposited to the ProteomeXchange Consortium *via* the PRIDE (Perez-Riverol et al., 2019) partner repository with the dataset identifier PXD024393.

### ^13^C Labeled Proteomics

Proteomics was carried out on the stirred bioreactor cyanobacterial consortia. 2.0 mL of consortia was transferred from 2 L stirred bioreactors into 100 mL glass bottles containing 50 mL of culture media in duplicate. 2.68 mL of 0.1 M NaH^13^CO_3_ was added for a total concentration of 0.536 M NaHCO_3_ containing 2% ^13^C (including the ∼1% natural abundance, background ^13^C). Cultures were subjected to day/night cycles of 16:8 h, using full spectrum LED lights (Model T5H0; 6400K, Sunblaster Holdings ULC, Langley, BC, Canada). The initial sample was taken directly from the 2 L stirred bioreactor. Time course samples were taken at 14 h (end of day 1), 22 h (end of night 1), 36 h (end of day 2), and 44 h (end of night 2). The 1 mL sample was centrifuged, supernatant media was removed, and the resulting pellet was frozen at −80°C for ∼10 days until protein extraction. Protein extraction was carried out as described above.

Extracted peptides were separated by an RSLC-nano Liquid Chromatograph (Thermo Fisher Scientific, Waltham, MA, United States), and subsequently analyzed on a QExatcive Plus hybrid quadrupole Orbitrap mass spectrometer (Thermo Fisher Scientific). Quadruplicate technical replicates were run as described above. Identification and quantification of detected proteins were carried out in Proteome Discover version 2.2.0.388 (Thermo Fisher Scientific) using the SEQUEST HT node. FDR were estimated at the peptide and protein level with Percolator Node and FidoCT, respectively and proteins and peptides with FDR > 5% discarded from further analysis. Proteins without protein-unique peptides were discarded. Protein database consisted of metagenome assembled genomes from the cyanobacterial consortia in this study. The ^13^C/^12^C of detected peptides were determined with Calis-p 2.0 software (Kleiner et al., 2021), using scored peptide spectra match (PSM) tables and raw MS data (in mzML format) as input^3^.

The ^13^C/^12^C ratio for each peptide mapping to a single MAG was attributed to the Taxonomy for that MAG. For peptides mapping to multiple MAGs the ratio was attributed to the most specific taxonomic level common to the protein taxonomic assignments. E.g., Peptides assigned to *Verrucomicrobiota, Opitutales* consist of MAGs 7A and 56, peptides assigned to *Verrucomicrobiota, Kritimatiellae* consist of MAGs 17 and 41, and peptides assigned to *Saccharospirillaceae* consist of MAGs 5A and 23.

The ^13^C/^12^C ratio for each taxon at a given time point was calculated as the median ratio of all peptides mapping to a given taxa weighted by the spectral intensity of that peptide in that sample using Hmisc 3.6.3 in R 3.6.2. Weighting of the ratio by spectral intensity accounts for the amount of that peptide detected, better reflecting the abundance of peptides with a given ratio. For cyanobacteria and heterotrophs (all peptides not mapped to *Cyanobacteria*), boxplots show the median ^13^C/^12^C ratio with upper and lower quantiles. For individual MAGs, in addition, individual points showing the ^13^C/^12^C ratio for each detected peptide at each time point were plotted to visualize the distribution and number of peptides detected. The mass spectrometry proteomics data have been deposited to the ProteomeXchange Consortium *via* the PRIDE (Perez-Riverol et al., 2019) partner repository with the dataset identifier PXD028578.

## Results and Discussion

### Metagenomics Reveals the Overall Stability of the Consortium

Previously, we enriched a cyanobacterial consortium in laboratory photobioreactors (PBRs) (Ataeian et al., 2019) from microbial mats of four alkaline soda lakes located on Cariboo Plateau, BC, Canada (Brady et al., 2013; Zorz et al., 2019). Recently, we introduced *Candidatus* “Phormidium alkaliphilum,” the most abundant member and the only cyanobacterium in the consortium (Ataeian et al., 2021). Here, we focus on the roles of the heterotrophic members of the consortium. For this, we made use of five metagenomes, collected from different cultures and time points as previously described (Ataeian et al., 2021). Two cultures had an initial pH of 8.3 (low-pH), one received ammonium as the nitrogen source, the other nitrate. Two cultures had an initial pH of 10.4 (high-pH), one received nitrate as the nitrogen source, the other urea. These cultures were maintained as biofilms in tubular PBRs. The fifth metagenome was obtained 2 years later from a stirred PBR. In this final PBR, the culture grew at an initial pH of 10.4, as small flocs or aggregates. This culture was seeded from the high-pH-nitrate culture. The medium of all five cultures always remained the same containing 0.5 mol/L of combined bicarbonate and carbonate at a starting pH of 8.3 or 10.4. During 5 days of growth, the pH increased, up to 11.2 in the high-pH experiments. No culture crashes were ever observed over the years of cultivation and the system showed robust and rapid growth with 15.2 ± 1.0 g/m^2^/day biomass productivity (Sharp et al., 2017; Ataeian et al., 2019).

To compare the community structure of the consortium for all five cultures, we used phyloFlash to reconstruct full length small subunit ribosomal RNA (SSU/16S rRNA) gene sequences from short Illumina reads (Gruber-Vodicka et al., 2019). The obtained full length SSU rRNA sequences enable taxonomic classification of the most abundant members of the consortium. This way, we determined the community structure for each of the five PBSs. In the low-pH tubular PBRs, >72% of the SSU rRNA reads recovered by phyloFlash were associated with *Candidatus* “Phormidium alkaliphilum.” This number increased to >87% in high-pH tubular PBR. After 2 years of growth in the stirred PBR at high-pH, 76.3% of the SSU rRNA reads were assigned to *Ca.* P. alkaliphilum. The remainder of the communities was associated with 28 Bacterial and 5 Eukaryote populations. The heterotrophs were classified to *Bacteroidota, Deinococcota, Gemmatimonadota, Planctomycetota, Proteobacteria*, and *Verrucomicrobiota* (Figure 1 and Supplementary Table 1). The Eukaryotic species were all affiliated with *SAR* (S*tramenopiles, Alveolates*, and *Rhizaria*) clade.

The apparent low diversity of the stirred culture is an artifact caused by the much lower sequencing coverage of its metagenome (Ataeian et al., 2021; Supplementary Table 3). Because of the lower number of reads, fewer complete 16S rRNA genes were assembled. For biodiversity analysis, reads from the stirred PBR were mapped to all the complete 16S rRNA genes collected from tubular PBRs.

At high pH, diversity among heterotrophs was lower, and less redundancy was observed within species of the same class (10 heterotrophic species were detected in high-pH cultures while 18 species were detected in low-pH cultures) (Supplementary Table 1). The abundance of *Bacteroidia, Deinococcota, Gemmatimonadota*, and *Alveolata* was higher at high-pH. *Gammaproteobacteria* were more abundant at low-pH (Figure 1 and Supplementary Table 1). Five eukaryotic species were detected. A species related to S*chmidingerothrix* from the order *Ciliophora* showed high abundance at high-pH (Figure 1 and Supplementary Table 1). S*chmidingerothrix* is a slender hypotrich ciliate which prefers a saline environment and feeds on bacteria (Foissner, 2012). Two species related to *Blastocystis* and *Halocafeteria* were more abundant at low-pH (Figure 1 and Supplementary Table 1). *Blastocystis* is a unicellular protist known to occur in the intestines of animals (Tan, 2008). *Halocafeteria* is a heterotrophic nanoflagellate frequently observed in hypersaline environments and feeds on prokaryotes (Park et al., 2006).

### Niche Partitioning Among Heterotrophic Consortium Members

**FIGURE 2:**
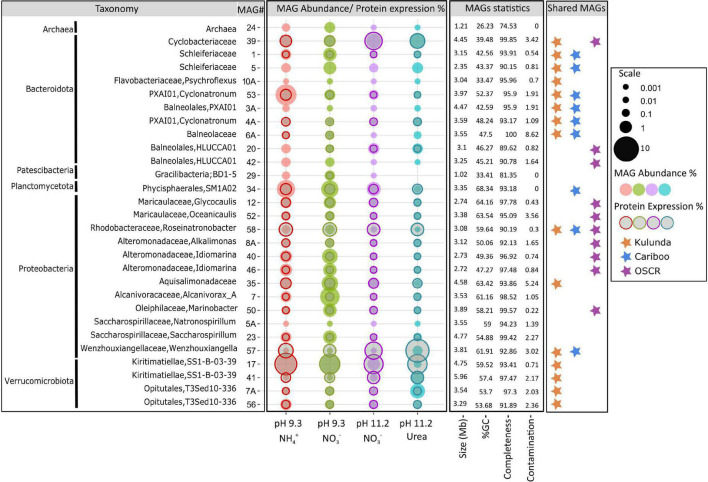
Taxonomy, relative abundance, and protein expression of the heterotrophic MAGs obtained from the *Candidatus* “Phormidium alkaliphilum” consortium. Taxonomic classifications are according to GTDBtk. Bubble plot showing abundances based on DNA sequencing and proteomics in four experiments with different pH and nitrogen sources. MAGs clustering at either family or genus level with Cariboo and Kulunda soda lakes MAGs as well as MAGs from the previously described *Phormidium* OSCR consortium are shown with stars. Data in Supplementary Tables 1, 2.

After comparing community structures with phyloFlash, we assembled and binned sequencing reads from all four 2018 metagenome samples to obtain MAGs of the most abundant (Supplementary Table 1) species of the consortium. We recovered 30 MAGs with completeness >90% (*n* = 27) and contamination <4% (*n* = 28). The GTDB-Tk (Parks et al., 2018) was used for taxonomic classification of all the MAGs (Figure 2 and Supplementary Table 2). In addition to the Cyanobacterium, *Ca.* P. alkaliphilum, each community was composed of at least 10 *Bacteroidota*, 3 *Alphaproteobacteria*, 9 *Gammaproteobacteria*, 4 *Verrucomicrobiota*, 1 *Patescibacteria*, 1 *Planctomycetota*, and 1 Archaea (Figure 2 and Supplementary Table 1). These classifications were consistent with phyloFlash results presented above, with three exceptions. One species of *Deinococcota* and one from *Gemmatimonadota* were binned into MAGs with completeness of less than 70% and were not analyzed further. Next, the 16S rRNA gene of the *Patescibacteria* MAG was not obtained by phyloFlash. Apart from that, all other MAGs could be matched to a 16S rRNA gene obtained from phyloFlash and the relative abundance profiles were fairly similar between the two analyses.

We also used GTDB-tk to assess the similarity of the PBR MAGs to 91 MAGs recently obtained from the original Cariboo mats (Zorz et al., 2019) and 871 MAGs from sediments of the Kulunda Steppe soda lakes (Figure 2 and Supplementary Table 2). Eight PBR MAGs clustered with both Cariboo and Kulunda lake MAGs at either family or genus level (Supplementary Figures 1–13) and were part of the core soda-lake microbiome identified by Zorz et al. (2019). Seven MAGs clustered only with Kulunda MAGs (Supplementary Figures 1–13). As the cultures were inoculated from Cariboo soda lakes, they were likely too rare in these lakes to be detected by Zorz et al. (2019). Two MAGs from *Bacteroidota*, seven from *Gammaproteobacteria*, two from *Alphaproteobacteria*, the only MAG from *Patescibacteria*, and the Archaea showed less similarity to Cariboo and Kulunda lake MAGs and clustered at either order or class level (Supplementary Figures 1–12). For these 12 MAGs, it is still unknown whether the associated species came from the soda lakes or joined the community during selective enrichment.

To further assess the overall stability of the consortium, we compared relative DNA sequence abundances and proteinaceous biomass contributions of each MAG and estimated the “turnover” of the heterotrophic community across conditions (Supplementary Table 1). Estimates based on relative DNA sequence abundance indicated that approximately 50% of the heterotrophic community was different among tubular PBR conditions and approximately 80% of the tubular PBR community was turned over (different) after 1 year in the stirred PBR. Estimates for turnover based on proteinaceous biomass contributions were lower, approximately 25% between tubular PBR conditions. There is no compelling reason to assign more confidence to protein or DNA based estimates, so the actual community turnover was estimated to be between 25 and 50% across tubular PBR conditions. Analysis of community turnover at different taxonomic levels (genus, family, order, and class) revealed slightly lower (44 versus 50% based on DNA, 20 versus 25% based on protein) turnover at class level, indicating that different taxa sharing the same class might fill in for each other to some extent (functional redundancy). Overall, the consortium’s heterotrophic community displayed a balance of conservation and dynamics within the context of the tubular PBRs. The shift to stirred PBRs provoked more rampant change within the heterotrophic community.

**FIGURE 3:**
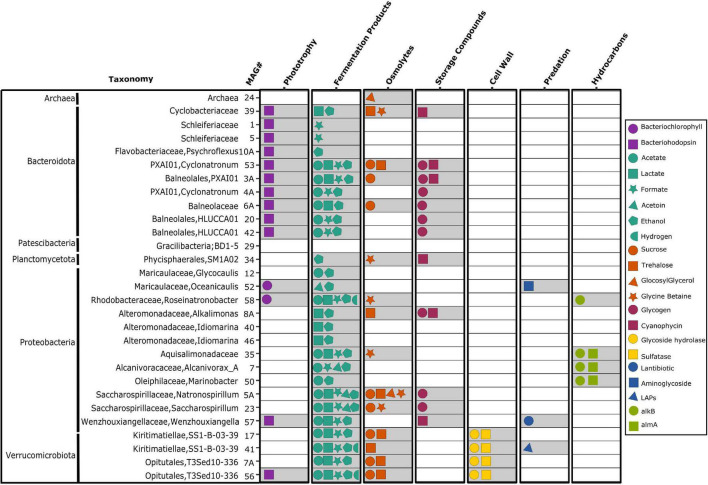
Inference of ecological niches based on gene content. For each MAG, inferred ecological niches are shown, with each symbol indicating a complete set of genes for the associated biochemical pathway. LAPs, linear azol(in)e-containing peptides; *alkB*, alkane-1-monooxygenase; and *almA*, flavin-binding monooxygenase. Detailed data in Supplementary Table 3.

The gene content of each MAG in the consortium was used to infer whether the consortium’s populations fulfilled the six ecological niches postulated in the introduction: (1) harvesting radiation not used by cyanobacteria as supplementary energy source; (2) use of cyanobacterial fermentation products; (3) use of cyanobacterial compatible solutes; (4) use of cyanobacterial storage compounds; (5) use of polysaccharides of the cyanobacterial cell wall; and (6) direct predation of other cells. For each carbon and energy source we looked for genes potentially involved in uptake and transport of the compound as well as for genes encoding biochemical pathways for their conversion. We also considered experimental studies of the phenotypes of related bacteria from the literature. Figure 3 shows the association of consortium’s populations with different ecological niches with details provided in Supplementary Table 3.

The ability to use light energy not used by cyanobacteria (niche 1) was found for half of the heterotrophic members of the consortium. MAG 52 (*Oceanicaulis*) and MAG 58 (*Roseinatronobacter*) from *Alphaproteobacteria* had genes for photosystem I, as well as for biosynthesis of bacteriochlorophyll *a* (Figure 3 and Supplementary Table 3). These systems are known to harvest wavelengths of light in the near infrared range of 800–850 nm (Croce and Van Amerongen, 2014). For comparison, cyanobacteria use chlorophylls as well as other antenna pigments to harvest visible light between 495 and 670 nm (Croce and Van Amerongen, 2014). The aerobic bacteriochlorophyll-containing bacteria perform cyclic electron flow, while translocating protons across the cytoplasmic membrane, powering ATP production (Ke et al., 1970; Kolber et al., 2000). *Oceanicaulis alexandrii* is closely related to MAG 52 (Supplementary Figure 2) and is known as non-motile rod-shaped aerobic chemoorganoheterotroph (Strömpl et al., 2003). *Roseinatronobacter monicus* is closely related to MAG 58 (Supplementary Figure 1) and is known as an alkaliphilic aerobic photoorganoheterotroph (Boldareva et al., 2007).

All consortium members affiliated with *Bacteroidota*, as well as MAG 57 (*Wenzhouxiangella*), and MAG 56 (*Verrucomicrobiota*) had genes for light-driven proton pumps known as proteorhodopsins (PRs) (Figure 3 and Supplementary Table 3; McCarren and DeLong, 2007; Pinhassi et al., 2016; Gómez-Consarnau et al., 2019). Phototrophy through proteorhodopsins can provide a considerable amount of energy (Gómez-Consarnau et al., 2007) for ATP synthesis (Steindler et al., 2011), vitamin B1 acquisition (Gómez-Consarnau et al., 2016), and bacterial survival during starvation (Gómez-Consarnau et al., 2010). Proteorhodopsins were found in various marine and freshwater ecosystems and harvest light at two different wavelengths. The green-absorbing pigments have a maximum absorption at 520 nm and the blue-absorbing pigments, typically found in deeper waters, have a peak absorption at 480 nm (Béjà et al., 2001). Proteorhodopsin so far has been detected in diverse bacterial groups including *Alphaproteobacteria* (Giovannoni et al., 2005; Moran and Miller, 2007), *Gammaproteobacteria* (Stingl et al., 2007), *Bacteroidetes* (Gómez-Consarnau et al., 2007), and *Verrucomicrobiota* (Martinez-Garcia et al., 2012). Members of the phylum *Bacteroidota* are known to grow attached to particles. They produce peptidases, receptors, and transporters for high-molecular-weight compounds and are best known for degradation of biopolymers, such as polysaccharides (Gómez-Pereira et al., 2012; Fernández-Gómez et al., 2013). Part of the energy to utilize these compounds could be provided through proteorhodopsins.

With regard to the use of cyanobacterial fermentation products (niche 2), almost all members of the consortium could use at least one of acetate, lactate, formate, acetoin, ethanol, and hydrogen (Figure 3 and Supplementary Table 3). This is in line with the capability of *Ca.* P. Alkaliphilum to produce all these fermentation products (Ataeian et al., 2021). More than half of the consortium members were capable of using osmolytes (niche 3) such as sucrose, trehalose, glucosylglycerol, and glycine betaine (Figure 3 and Supplementary Table 3). Although *Ca.* P. Alkaliphilum was shown to be capable of producing many osmolytes, the ability to produce glycine betaine was not detected (Ataeian et al., 2021). However, genes for glycine betaine biosynthesis were detected in consortium members affiliated with *Proteobacteria* (MAGs 12 and 52) and *Verrucomicrobiota* (MAGs 7A and 56).

The ability to use storage compounds glycogen or cyanophycin as carbon and nitrogen source was postulated as niche 4. Many bacteria can accumulate an intracellular glycogen which can serve as an energy source during carbon limitation. However, only few bacteria are able to use exogenous glycogen (Abbott et al., 2010). All consortium members affiliated with the order *Balneolales* as well as three MAGs from *Gammaproteobacteria* (MAGs 8A, 5A, and 23) encoded the genes for pullulanase (Figure 3 and Supplementary Table 3). This cell surface-anchored debranching exoenzyme facilitates the depolymerization and exogenous utilization of glycogen (Bender and Wallenfels, 1966; Bongaerts et al., 2000; Abbott et al., 2010). *Natronospirillum operosum*, closely related to MAG 5A and MAG 23 has been isolated from decaying biomass of a laboratory culture of cyanobacterium *Geitlerinema* sp. with obligately alkaliphilic growth (pH range 7.3–10.4) (Kevbrin et al., 2020). Growth on various carbohydrates, amino acids and proteinaceous substances has been shown for this species consistent with multiple niches associated with these MAGs. Cyanophycin utilization was detected in six members of the consortium from various phyla, all encoding cyanophycinase.

All four consortium members affiliated with *Verrucomicrobiota* had 6–38 genes for glycoside hydrolysis and 12–37 genes encoding sulfatases (Figure 3 and Supplementary Table 3). This indicated that they may use sulfated glycopolymers as their carbon and energy source (niche 5). Genome sequencing studies showed that a large number of sulfatase genes is one of the features of the *Planctomycetes–Verrucomicrobia–Chlamydia* (PVC) superphylum (Thrash et al., 2010; Wegner et al., 2013; Spring et al., 2016). Growth experiments on various sulfated polysaccharides followed by gene expression profiles of a marine *Planctomycete* showed that sulfatases are required for degradation and subsequent utilization of sulfated glycopolymers as a source of carbon (Glöckner et al., 2003; Wegner et al., 2013). Many cyanobacteria have been shown to produce sulfated exopolysaccharides, with many species containing thick capsules surrounding the cells (Vincenzini et al., 1990; De Philippis et al., 1998). Recently, genomic and proteomic analysis of a *Verrucomicrobiota* bacterium showed that this species specializes in consumption of sulfated polysaccharides containing methyl pentoses during spring algal blooms in the North Sea (Orellana et al., 2021). These findings show that PVC members are likely capable of consumption of sulfated glycopolymers using glycoside hydrolases and sulfatases. Lack of genes for biogenesis of a pilus or flagellum in the *Verrucomicrobiota* members of the consortium suggested a non-motile lifestyle. This could indicate their possible attachment to the cyanobacterial cell walls or sheath, while feeding on sulfated glycopolymers.

Direct predation of other cells (niche 6) was investigated by a comprehensive search for gene clusters encoding antimicrobial functions. AntiSMASH identified three consortium members, MAG 57 (*Wenzhouxiangella*), MAG 52 (*Oceanicaulis*), and MAG 41 (*Kiritimatiellae*), to be possible predators (Figure 3). The *Wenzhouxiangella* MAG contains a gene cluster encoding lantibiotic biosynthesis proteins, as was also found previously in a related species isolated from alkaline soda lakes in the Kulunda Steppe (Sorokin et al., 2020). Lantibiotics are post-translationally modified antimicrobial peptides causing cell death, possibly by disturbing the cytoplasmic membrane (van Kraaij et al., 1999). This could lead to the partial disintegration of the target cells. MAG 52 contained core biosynthetic genes for aminoglycoside biosynthesis. Aminoglycosides are bactericidal antibiotics previously shown to target aerobic, gram negative bacteria. They function by creating fissures in the outer membrane of the cell and inhibit protein synthesis through binding to ribosomes (Hancock, 1981; Mingeot-Leclercq et al., 1999). Core biosynthetic genes for another group of post-translationally modified peptides, linear azol(in)e-containing peptides (LAPs), were detected in one of the two consortium members affiliated with *Kiritimatiellae*. LAPs have different antibacterial activities, such as disturbing the cell envelope (Molohon et al., 2016) or inhibiting ribosomal function (Metelev et al., 2017).

It remains unknown which cells are targeted by the predators. Given that MAG57 and MAG41 were among the most abundant heterotrophs, the cyanobacterium itself might be a possible target. However, 4 years of crash-free, productive growth indicated that if the cyanobacterium was targeted, some factor must have limited the effectiveness of these predators. Alternatively, the presence of predatory consortium members may create an additional barrier to successful colonization by invaders, in addition to resource depletion. Invading microbes might lack resistance to these antimicrobials, because of the lack of prior exposure. For example, *Pseudoalteromonas tunicata* resides on the surface of the marine alga *Ulva lactuca* and the *tunicate Ciona intestinalis* and produces a range of antifouling metabolites, protecting its host. Antibacterial proteins (James et al., 1996), anti-larval molecules (Maki et al., 1988), antialgal peptides (Egan et al., 2001), antifungal tambjamine molecules (Franks et al., 2006), and violacein (Matz et al., 2008) are some of the targeted chemical defense mechanisms used by *P. tunicata*.

The presence of bacteria related to well-known hydrocarbon degrading species such as *Alcanivorax* (MAG 7) and *Marinobacter* (MAG 50) brought up the possibility of hydrocarbon degradation as an unforeseen niche, defined *a posteriori* (niche 7). Some cyanobacteria are known to co-exist with hydrocarbon degrading bacteria such as *Alcanivorax* and *Marinobacter* (Green et al., 2004, 2015; Yakimov et al., 2007; McGenity et al., 2012). In the absence of hydrocarbons, *Alcanivorax* can still grow with substrates such as pyruvate and succinate (Fernández-Martínez et al., 2003; Naether et al., 2013). However, in our consortia, they would likely be outcompeted by other heterotrophic bacteria. *Alcanivorax* spp. has been shown to use alkanes released by marine cyanobacteria (Coates et al., 2014; Lea-Smith et al., 2015) and other hydrocarbon-producing eukaryotic algae (Sorigué et al., 2016) using alkane-1-monooxygenase (*alkB*) and flavin-binding monooxygenase (*almA*). Two marine cyanobacteria, *Prochlorococcus* and *Synechococcus*, produce and accumulate C15 and C17 alkanes through two separate pathways (Schirmer et al., 2010; Mendez-Perez et al., 2011; Lea-Smith et al., 2015). One pathway comprises a two-step conversion of fatty acids to fatty aldehydes and then alkanes using fatty acyl ACP reductase (FAAR) and aldehyde deformylating oxygenase (ADO). The second involves elongating the acyl chain by a polyketide synthase (PKS) followed by decarboxylation to produce a terminal alkene. *Ca.* P. alkaliphilum synthesizes long chain alk(a/e)nes using FAAR and ADO through the first pathway. Protein expression for FAAR and ADO has been shown in Ataeian et al. (2021). The ability to degrade hydrocarbons produced by *Ca.* P. alkaliphilum was assessed in the heterotrophic members of the consortium. The genes *alkB* and *almA* were detected in both MAG 7 (*Alcanivorax*) and MAG 50 (*Marinobacter*) as well as in MAG 35 (*Aquisalimonadaceae*) and MAG 58 (*Roseinatronobacter*) (Figure 3). Thus, use of hydrocarbons was discovered as a seventh niche supported in this cyanobacterial consortium.

Although we did not define CO_2_ fixation as a possible niche for heterotrophs, genes for inorganic carbon fixation were detected within MAG 35 (*Aquisalimonadaceae*). All of the genes involved in the Calvin–Benson Cycle (CBC), including both subunits of form I ribulose 1,5-bisphosphate carboxylase (RuBisCO), were identified (Supplementary Table 3). Genes encoding a nitrate reductase complex indicate this organism might be a facultative nitrite oxidizer. Alternatively, this bacterium might use the CBC cycle as a sink for reducing equivalents produced during heterotrophic metabolism. This bacterium was otherwise assigned to use of fermentation products, osmolytes, and storage compounds.

Most heterotrophic populations of the consortium were found to occupy multiple ecological niches, and most niches were filled by more than one consortium member, demonstrating functional redundancy among species. None of the discussed niches could be detected in *Gracilibacteria*, *Patescibacteria*, the only species affiliated with Candidate Phyla Radiation, MAG 29. Genomic analysis of the members of this phylum has shown that they have limited metabolism sometimes lacking glycolysis, the pentose phosphate and Entner-Doudoroff pathways. They likely live a symbiont or endosymbiont lifestyle (Sieber et al., 2019). They have been shown to acquire pyruvate, acetyl coenzyme A (acetyl-CoA), and oxaloacetate through degradation of externally derived citrate, malate, and amino acids (Sieber et al., 2019). Given its low abundance, it remains unknown which other consortium member functions as its partner or host.

The one Archaea, affiliated with *Nanoarchaeia* (Nanoarchaeota phylum, MAG 24), in the consortium, contained genes only for glucosylglycerol degradation among the seven niches (Figure 3). The only representative from this phylum, *Nanoarchaeum equitans*, was shown to be an obligate symbiont, lacking genes for lipid, cofactor, amino acid, or nucleotide biosynthesis, but encodes genes for information processing and repair (Waters et al., 2003; Castelle et al., 2018).

### Stable Isotope Probing/Proteomics Shows Uptake of Carbon Sources by Community Members During Day and Night

To determine the carbon transfer dynamics in the consortium we used high-throughput stable isotope probing with ^13^C-labeled bicarbonate, followed by proteomics (protein/SIP). This way, we determined the ^13^C/^12^C ratio for individual populations based on the ^13^C content of their peptides. Experiments were carried out over 2 day/night cycles. The analytical error in the protein/SIP results is mainly determined by the number of peptides analyzed. Because most heterotrophs were relatively rare and yielded only few peptides for analysis, the estimates for their ^13^C content often remained quite rough. This made quantitative interpretation of dynamics difficult.

**FIGURE 4:**
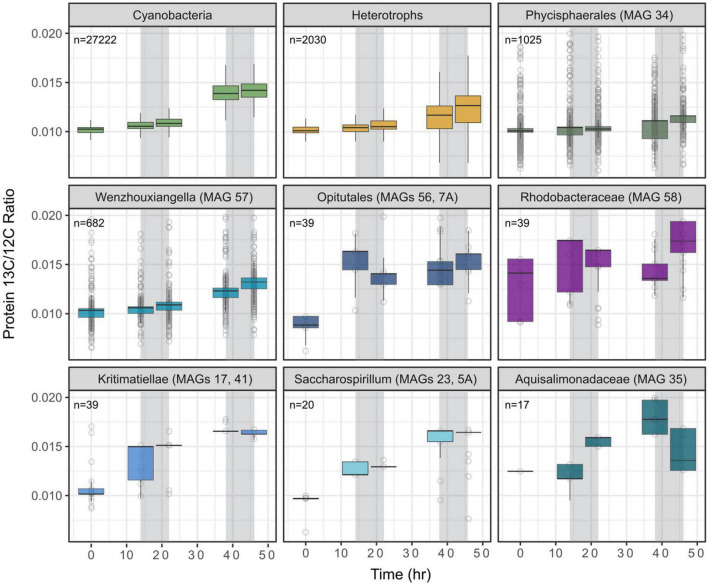
Carbon flow from *Candidatus* “Phormidium alkaliphilum” (Cyanobacteria) to the consortium’s heterotrophs, assessed by SIP/Proteomics. The culture was incubated with 2% ^13^C for 4 days. Gray shadings indicate the 8 h dark periods. Box plots show weighted mean ^13^C/^12^C ratio (middle bar) and 25 and 75% confidence intervals. Individual peptide ratios and number of peptides assigned are indicated. Supplementary Table 4 provides summary statistics.

The box and whisker plots and trends presented in Figure 4 shows uptake of ^13^C by *Ca.* P. alkaliphilum and, potentially, transfer to the heterotrophic populations. *Ca.* P. alkaliphilum showed the highest number of peptides with measurable ^13^C content (*n* = 27,222). The aggregated heterotrophic signal was dominated by two heterotrophs that were most abundant in this experiment: MAG 34 *Phycisphaerales* (MAG 34) and *Wenzhouxiangella* (MAG 57). The five heterotrophs detected in lower abundance (<40 peptides) *Opitutales* (MAGs 56 and 7A), *Rhodobacteraceae* (MAG 58), *Kiritimatiellae* (MAGs 17 and 41), *Saccharospirillum* (MAGs 23 and 5A), and *Aquisalimonadaceae* (MAG 35), do show evidence for ^13^C uptake, but the dynamics were hard to interpret. These populations were all still among the most abundant heterotrophs at high-pH (Figure 2 and Supplementary Table 1).

During day 1 and night 1, the median ^13^C/^12^C ratio of *Ca.* P. alkaliphilum showed only a modest increase (Figure 4 and Supplementary Table 4). During day 2, the rate of ^13^C assimilation into protein by *Ca.* P. alkaliphilum peaked, and the ^13^C/^12^C ratio increased by 3.0 × 10^–3^ over 16 h. The second night showed a slower increase at 0.2 × 10^–3^ over 8 h. The lower incorporation at night time compared to day time may indicate *Ca.* P. alkaliphilum mainly performs protein synthesis during day time, as previously observed (Singer and Doolittle, 1975; Hood et al., 2016). Additionally, the differences between the rate of ^13^C/^12^C increase during day 1 and day 2 suggest that C incorporation into cyanobacterial proteins is a two-step process: the newly fixed carbon first accumulates in an intermediate glycogen pool before being assimilated into proteins (Polerecky et al., 2021; Rabouille et al., 2021). Such an intermediate glycogen pool would initially consist mainly of ^12^C, as it was synthesized before the addition of ^13^C-bicarbonate. Our data indicate that ^13^C bicarbonate makes its way *via* the glycogen pool into the *Ca.* P. alkaliphilum protein pool on the timescale of a day.

The bulk heterotrophic ^13^C/^12^C ratio generally follows the same pattern as *Ca.* P. alkaliphilum, with the largest increase in ^13^C/^12^C on day 2, and more incremental increases at other timepoints. This is consistent with the assumed transfer of carbon from *Ca.* P. alkaliphilum to heterotrophic populations. Metabolic niche analysis indicated several possible mechanisms of uptake of fixed organic carbon, summarized in Figure 3. For the two most abundant heterotrophs, affiliated with *Phycisphaerales* (MAG 34) and *Wenzhouxiangella* (MAG 57), the increase in ^13^C/^12^C of their proteins lagged behind the increase observed for *Ca.* P. alkaliphilum, suggesting that these two heterotrophic populations derived their protein from *Ca.* P. alkaliphilum macromolecules (e.g., protein, glycogen). For the other heterotrophs shown in Figure 4, ^13^C uptake appeared to precede cyanobacterial uptake, suggesting that these populations derived their ^13^C-labeled proteins directly from *Ca.* P. alkaliphilum metabolites rather than ^13^C-labeled *Ca.* P. alkaliphilum protein. Cyanobacteria can release these so-called “leaky-metabolites,” such as pyruvate and 2-oxoglutarate, because high rates of carbon fixation can overwhelm their biosynthetic pathways (Cano et al., 2018). For example, cyanobacteria in marine surface waters release each day the same series of metabolites, which are subsequently assimilated or metabolized by heterotrophic bacteria (Aylward et al., 2015). Whether a similar trophic cascade occurred also in our PBR systems cannot be ascertained from the present data.

### Similar Cyanobacteria and Similar Consortia

At an average nucleotide identity (ANI) of 86.3%, *Phormidium* OSCR (GCA_001314905.1) (Nelson et al., 2016) is closely related to *Ca.* P. alkaliphilum (Ataeian et al., 2021). A functionally stable cyanobacterial consortium containing *Phormidium* OSCR was previously enriched from microbial mats in Hot Lake (Cole et al., 2014). The *P.* OSCR culture grows at Hot Lake conditions that include high concentrations of magnesium sulfate (400 mM). We used the GTDBtk to investigate the similarities between the *Ca.* P. alkaliphilum and *P.* OSCR consortia. Despite differences in medium composition, salinity, pH, and alkalinity of the systems (Supplementary Table 5), the two consortia exhibited fairly similar community structure. Both contained many heterotrophic populations even though no exogenous organic carbon source was provided. A total of 12/18 MAGs from *P. OSCR* consortium clustered together with 9 MAGs from *Ca.* P. alkaliphilum consortium at the family or genus level (Figure 2, Supplementary Table 2, and Supplementary Figures 1–13). The shared heterotrophic populations were MAG numbers 39, 20, 42, 52, 58, 8A, 40, 46, and 50 assigned to *Alphaproteobacteria*, *Gammaproteobacteria*, and *Bacteroidota*. This suggests *Ca.* P. alkaliphilum and *P. OSCR* support similar ecological niches. The shared heterotrophs occupy all the discussed niches except for utilization of sulfated glycopolymers of the cell wall. Probably, this niche was occupied by very different species in these two consortia.

### Potential Productivity Losses Associated With Heterotrophs

Growth of cyanobacterial photoautotrophs drives biomass production. This organic carbon supports growth of heterotrophic bacteria. The increase in the ^13^C/^12^C ratio of heterotrophic bacteria suggested transfer of carbon from cyanobacteria to heterotrophs and provided experimental support for this notion. As the aim of cyanobacterial biotechnology is to produce cyanobacteria, heterotrophic growth may be undesirable from a productivity perspective. For example, when growing cyanobacteria to produce phycocyanin, a valuable proteinaceous blue pigment, the presence of heterotrophs may reduce the overall phycocyanin yield.

In our consortium, the relative abundance of heterotrophs was 17 ± 7%, as estimated based on the combined metagenome, proteome, and phyloFlash data (Supplementary Table 1). Aerobic heterotrophs respire up to one additional carbon atom for every carbon atom they assimilate (Roller and Schmidt, 2015). Assuming that the relative abundance is a good estimator for the relative amount of biomass, the net cyanobacterial biomass production of ∼83 g is a result of the gross cyanobacterial biomass production of ∼117 g combined with a loss of ∼34 g of cyanobacterial biomass due to the growth of heterotrophs (∼17 g each for the heterotrophic anabolism and catabolism). Thus, the efficiency of the total biomass production in the consortium was about 85% (100/117) and the efficiency of the cyanobacterial biomass production was about 70% (83/117). For comparison, the overall energy conversion efficiency. For comparison, the overall energy conversion efficiency (from incident sunlight to biomass produced) of natural photosynthetic systems such as microbial mats or man-made algae cultivation systems ranges between 1 and 7% (Al-Najjar et al., 2010, 2012; Ooms et al., 2016; Zorz et al., 2021). The overall efficiency is low because of many factors, including reflection of light from the photobioreactor or water surface, partial utilization of the available light spectrum, light absorption by photosynthetically inactive molecules or particles, a relatively low maximum turnover rate of photosystems, losses during energy conservation and CO_2_ fixation (“dark”) reactions, and cell maintenance processes. Thus, losses associated with the conversion of light energy into the production of heterotrophic biomass are relatively minor. Also, it is unclear whether cyanobacterial pure cultures are able to convert all the produced carbon into useful biomass. Overflow of fermentation products (Cano et al., 2018), release of osmolytes (Hagemann, 2011), and remains of dead cells (Aguilera et al., 2021) are examples of organic carbon produced by cyanobacteria that may remain unused even in the absence of heterotrophs.

Heterotrophic members of the consortium were assigned to seven ecological niches: phototrophy, use of fermentation products, compatible solutes, storage compounds, polysaccharides of the cyanobacterial cell wall, direct predation, and hydrocarbons. Most niches were filled by more than one consortium member, demonstrating functional redundancy among heterotrophs. Occupation of heterotroph niches, along with redundancy within niches may explain the apparent ecological robustness of the consortium, which is evident from 4 years of crash-free growth in open laboratory culture, as well as the seamless adaptation to different culture conditions. The similar community structure of a consortium of a closely related cyanobacterium supported the occurrence of the same suite of ecological niches.

## Data Availability Statement

The datasets presented in this study can be found in online repositories. The names of the repository/repositories and accession number(s) can be found below: https://www.ncbi.nlm.nih.gov/, BioProject PRJNA377096, Biosamples SAMN21419665-93, SAMN17969417-20, and SAMN18025958-60; https://www.ebi.ac.uk/pride/archive/, PXD024393, PXD028578.

## Author Contributions

MA performed metagenome sequencing, data analysis as well as proteomics and data processing, and drafted the manuscript. YL, AK, and AH performed protein/SIP experiments and analyzed and interpreted the data. MS and AH reviewed and edited the manuscript. All authors approved the submitted version.

## Conflict of Interest

The authors declare that the research was conducted in the absence of any commercial or financial relationships that could be construed as a potential conflict of interest.

## Publisher’s Note

All claims expressed in this article are solely those of the authors and do not necessarily represent those of their affiliated organizations, or those of the publisher, the editors and the reviewers. Any product that may be evaluated in this article, or claim that may be made by its manufacturer, is not guaranteed or endorsed by the publisher.

## Footnotes

1
http://github.com/HRGV/phyloFlash

2
http://www.thegpm.org/crap/

3
https://sourceforge.net/p/calis-p/wiki/Home/
