# Supplementary material for: Ecological Interactions of Cyanobacteria and Heterotrophs Enhances the Robustness of Cyanobacterial Consortium for Carbon Sequestration
Source: Front Microbiol. 2022 Feb 11;13:780346. doi: 10.3389/fmicb.2022.780346 (PMC8880816; doi:10.3389/fmicb.2022.780346)
Supplement: Supplementary file 6 [file Data_Sheet_1.PDF]

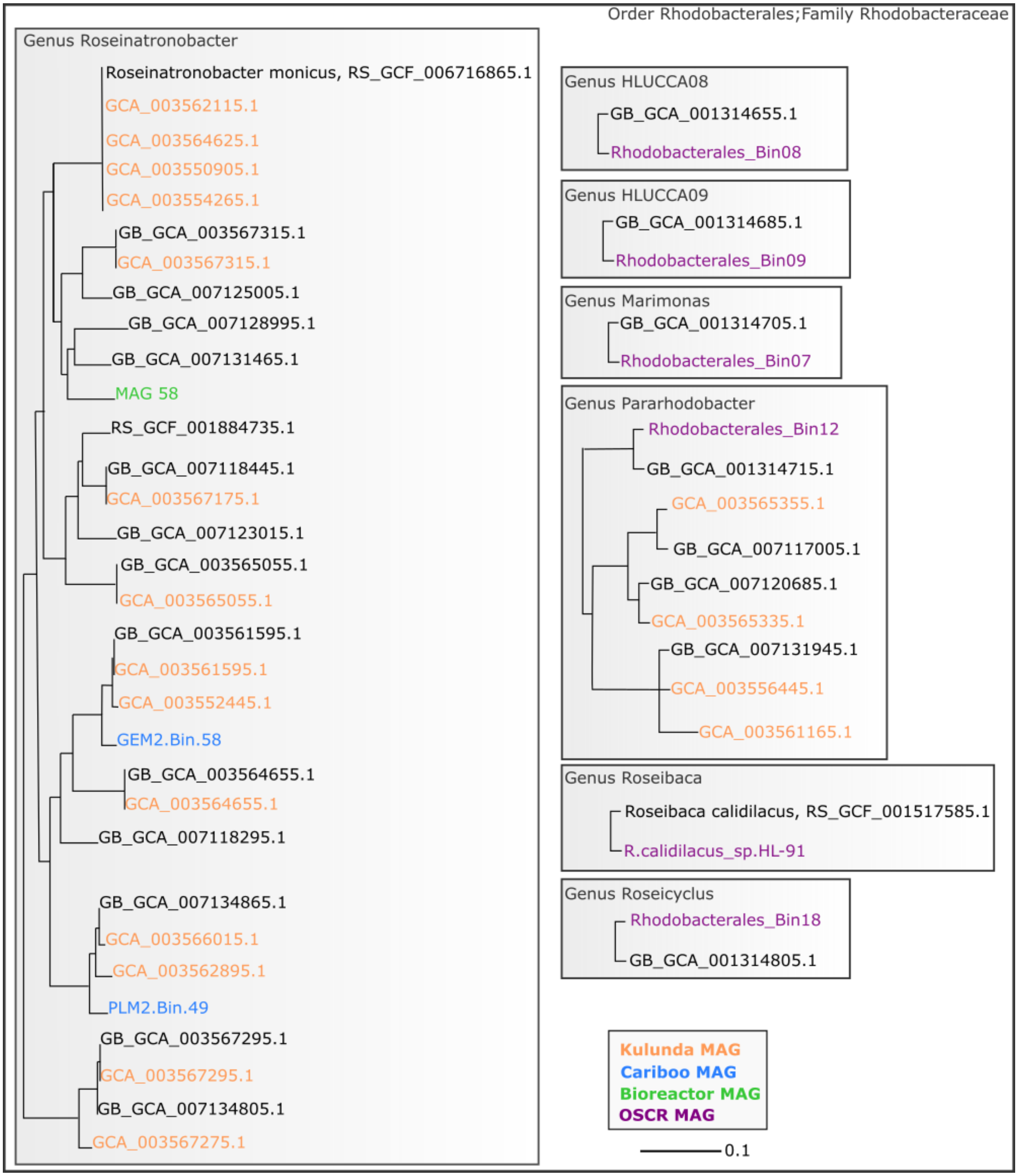

Figure S1 Relationship of the consortium's *Alphaproteobacteria* MAGs to MAGs directly obtained from Cariboo and Kulunda alkaline soda lakes as well as the previously described *Phormidium* OSCR consortium.

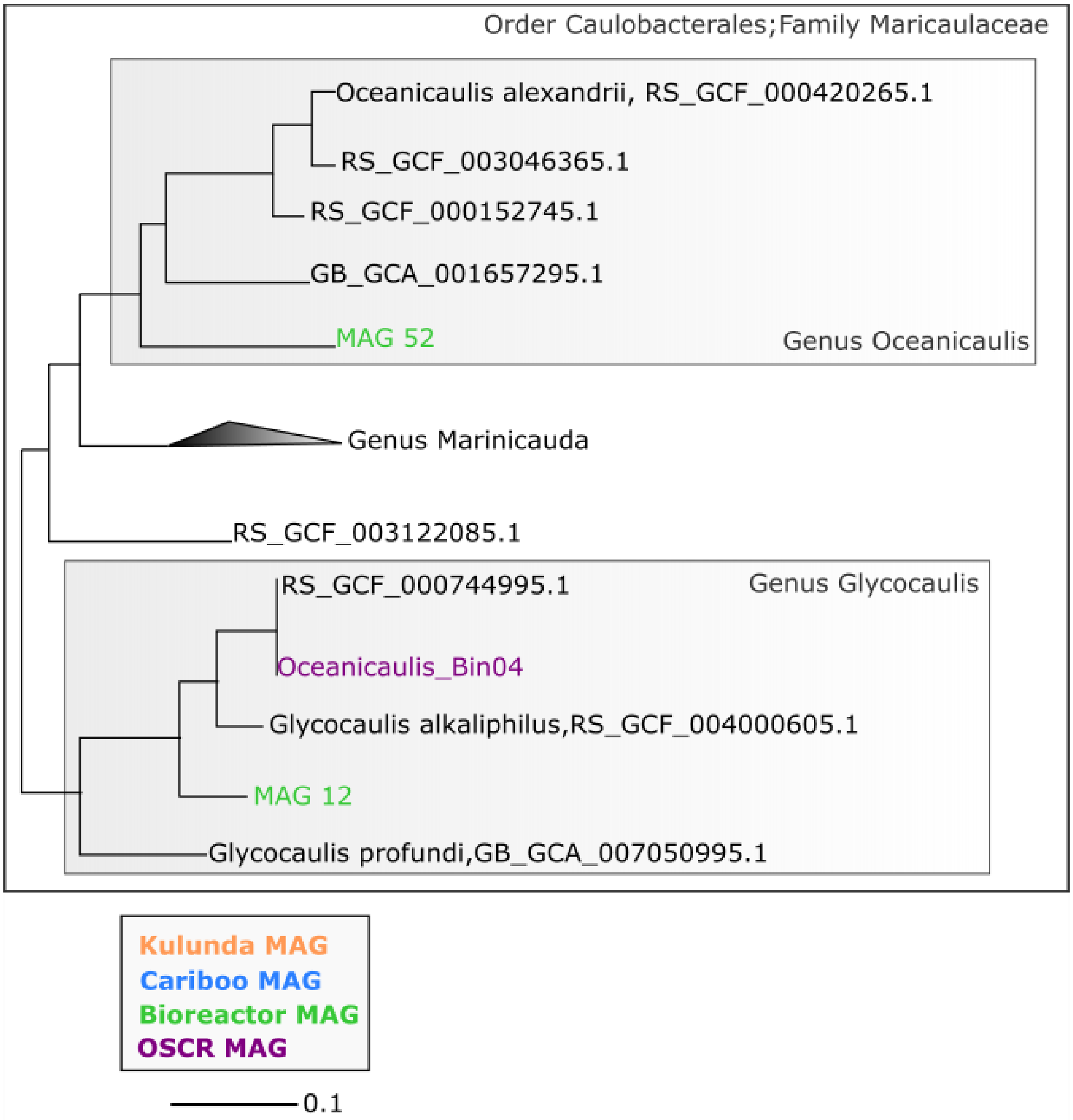

Figure S2 Relationship of the consortium's *Alphaproteobacteria* MAGs to MAGs directly obtained from Cariboo and Kulunda alkaline soda lakes as well as the previously described *Phormidium* OSCR consortium.

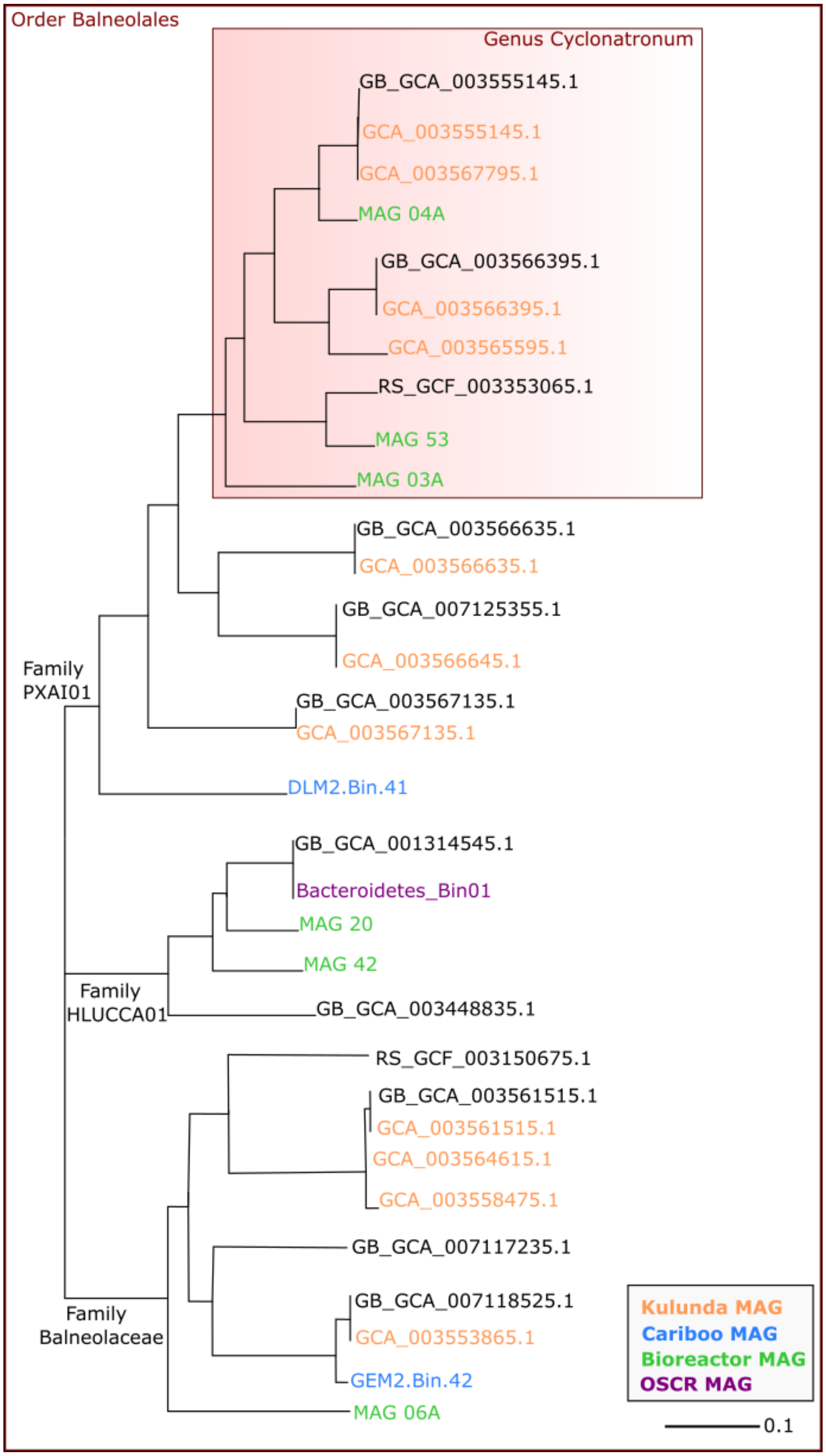

Figure S3 Relationship of the consortium's *Bacteroidota* MAGs to MAGs directly obtained from Cariboo and Kulunda alkaline soda lakes as well as the previously described *Phormidium* OSCR consortium.

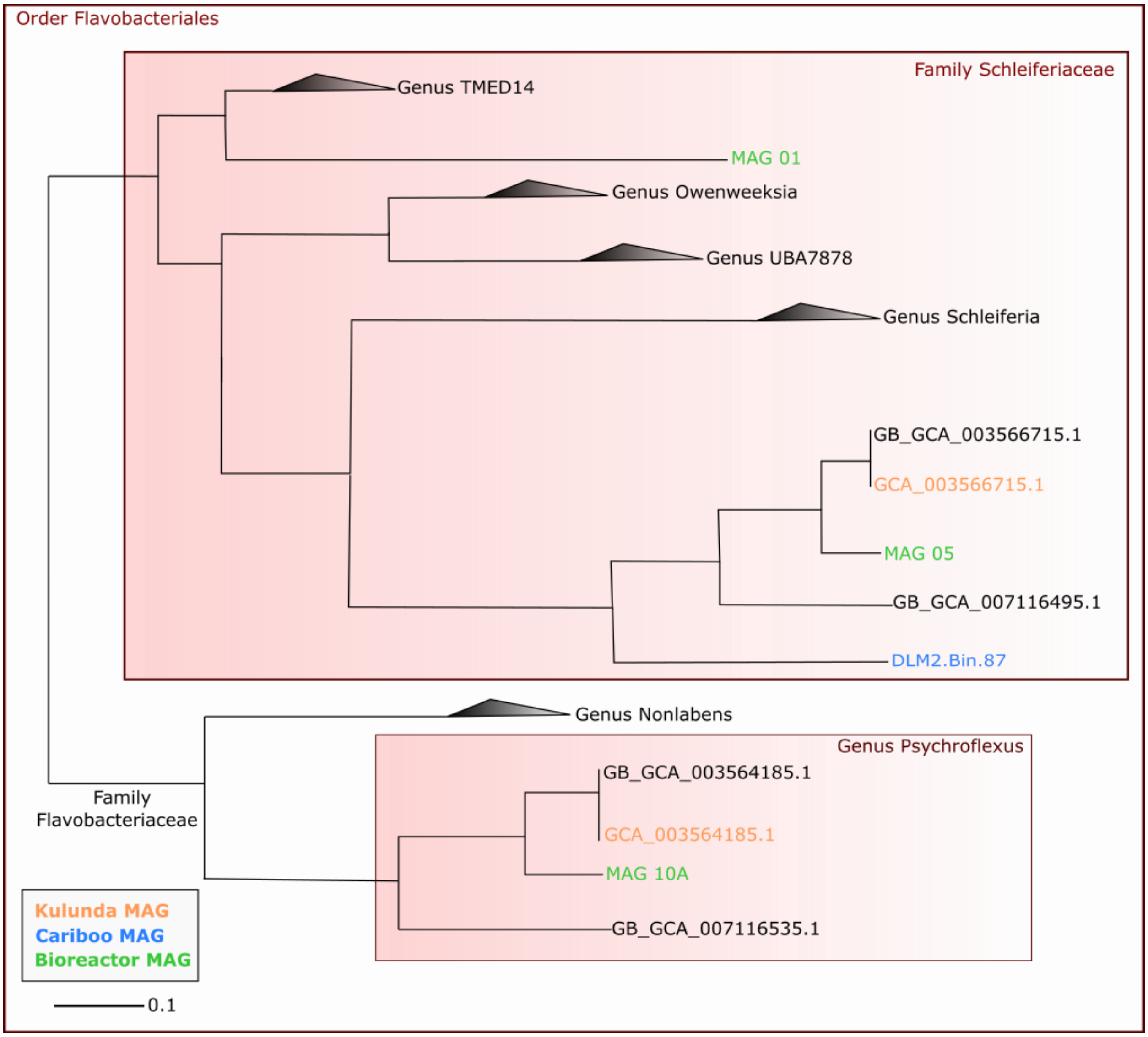

Figure S4 Relationship of the consortium's *Bacteroidota* MAGs to MAGs directly obtained from Cariboo and Kulunda alkaline soda lakes as well as the previously described *Phormidium* OSCR consortium.

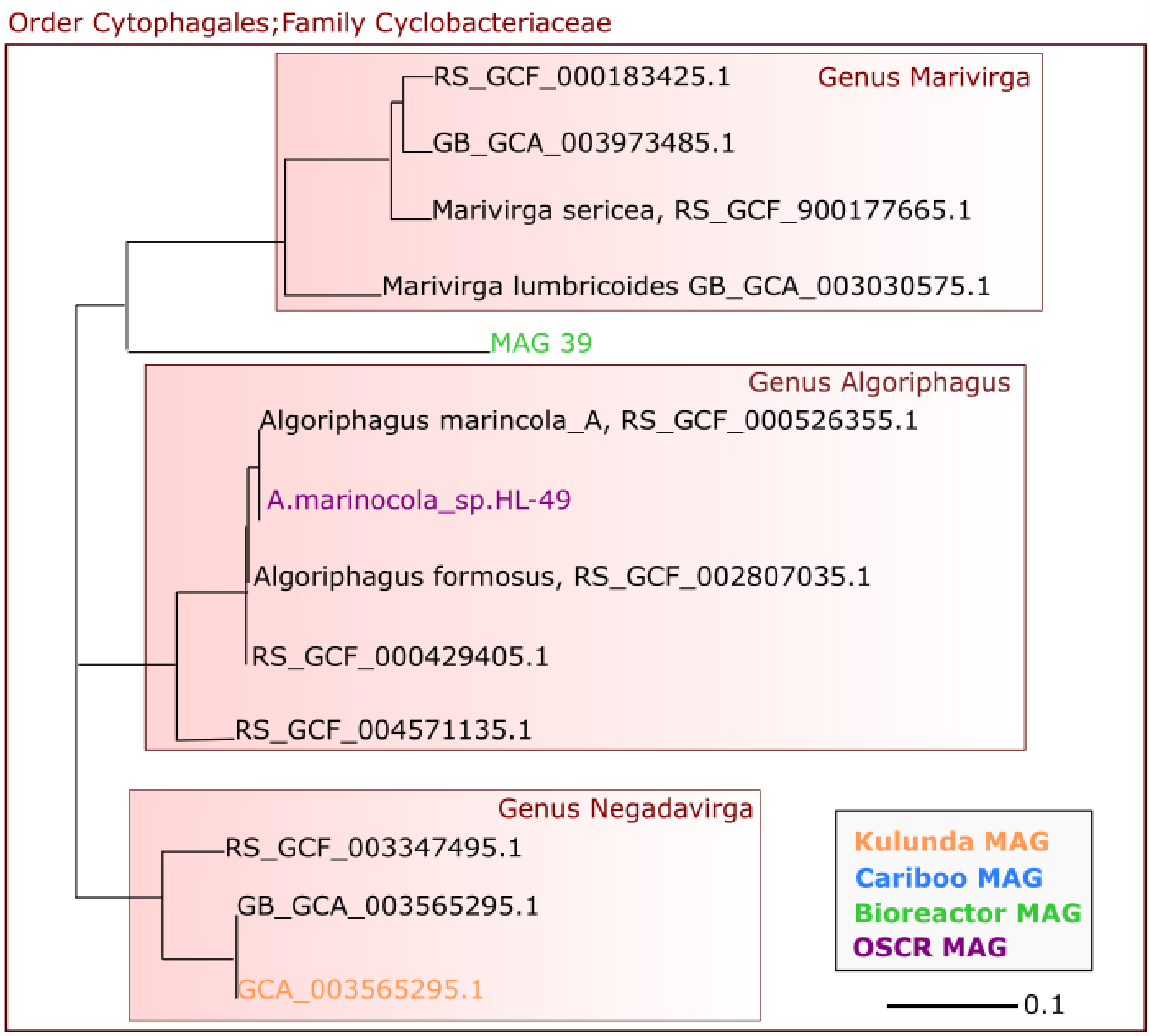

Figure S5 Relationship of the consortium's *Bacteroidota* MAGs to MAGs directly obtained from Cariboo and Kulunda alkaline soda lakes as well as the previously described *Phormidium* OSCR consortium.

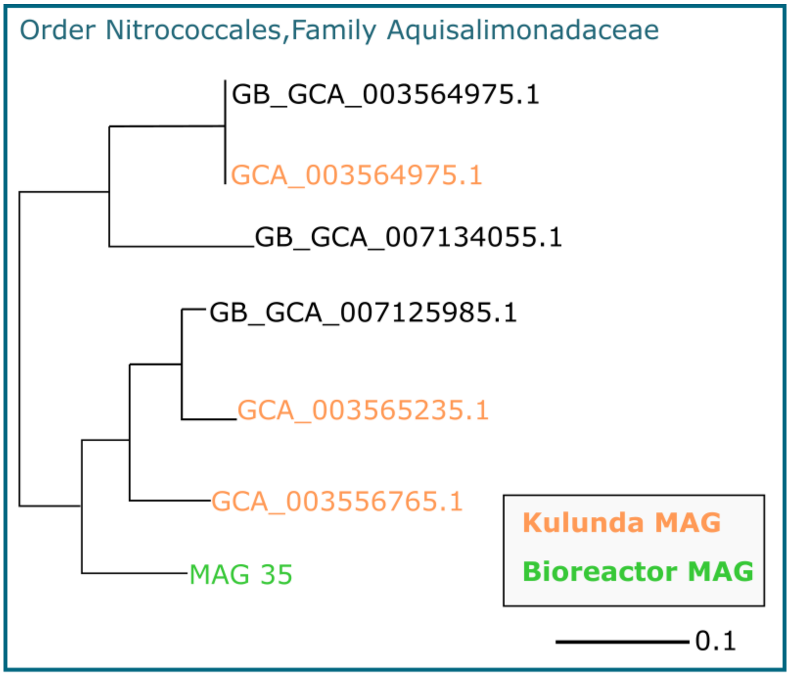

Figure S6 Relationship of the consortium's *Gammaproteobacteria* MAGs to MAGs directly obtained from Cariboo and Kulunda alkaline soda lakes as well as the previously described *Phormidium* OSCR consortium.

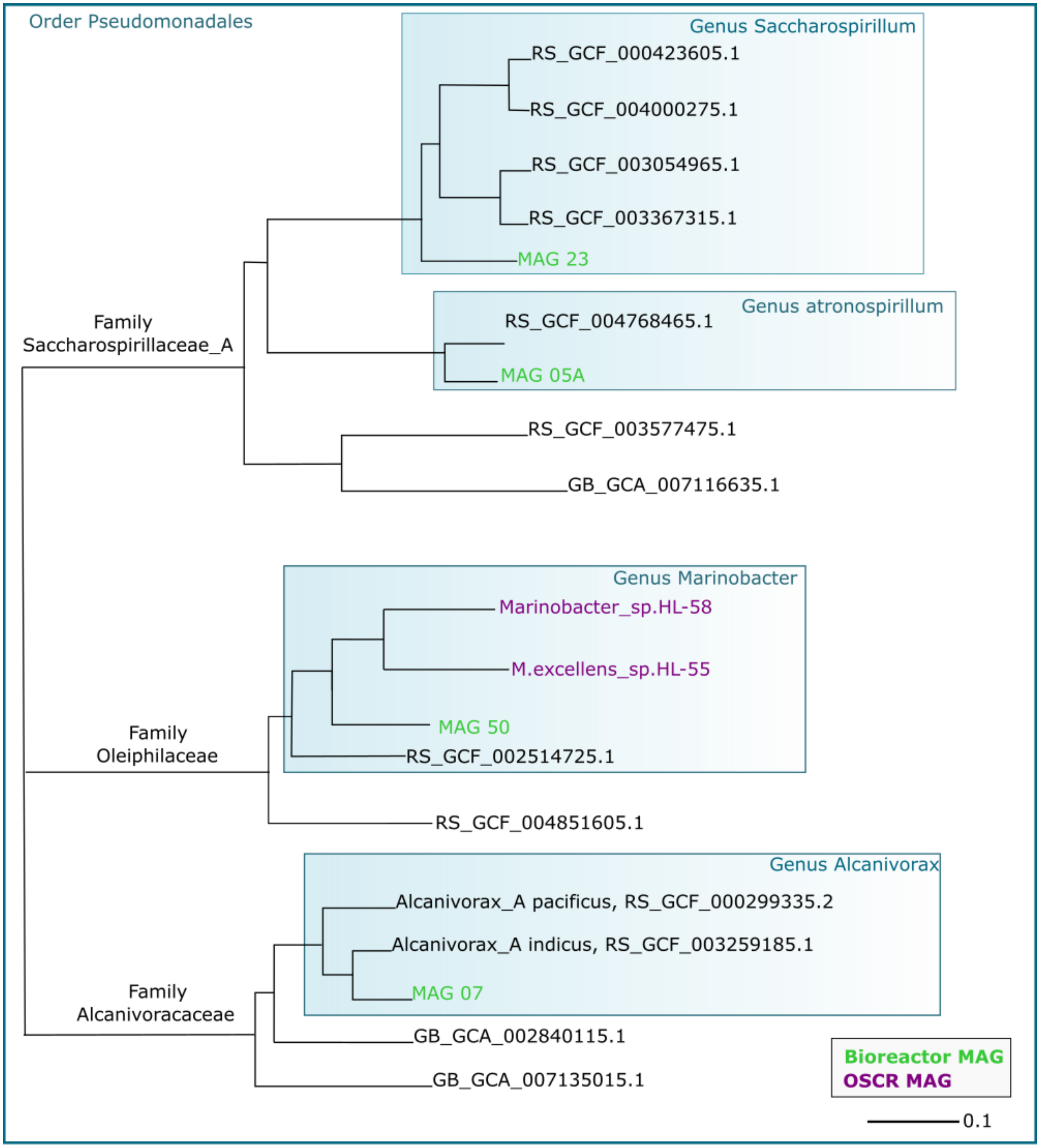

Figure S7 Relationship of the consortium's *Gammaproteobacteria* MAGs to MAGs directly obtained from Cariboo and Kulunda alkaline soda lakes as well as the previously described *Phormidium* OSCR consortium.

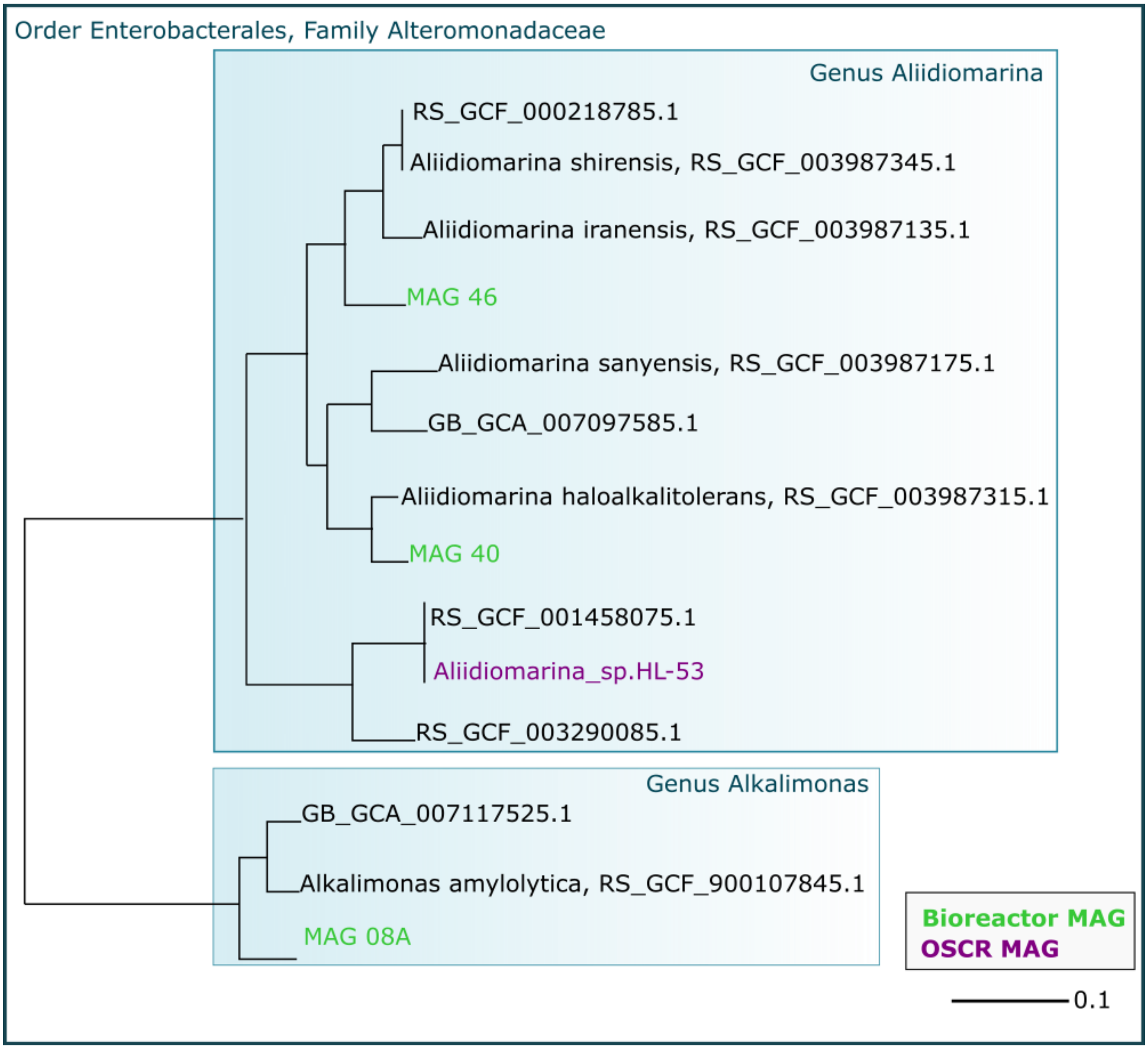

Figure S8 Relationship of the consortium's *Gammaproteobacteria* MAGs to MAGs directly obtained from Cariboo and Kulunda alkaline soda lakes as well as the previously described *Phormidium* OSCR consortium.

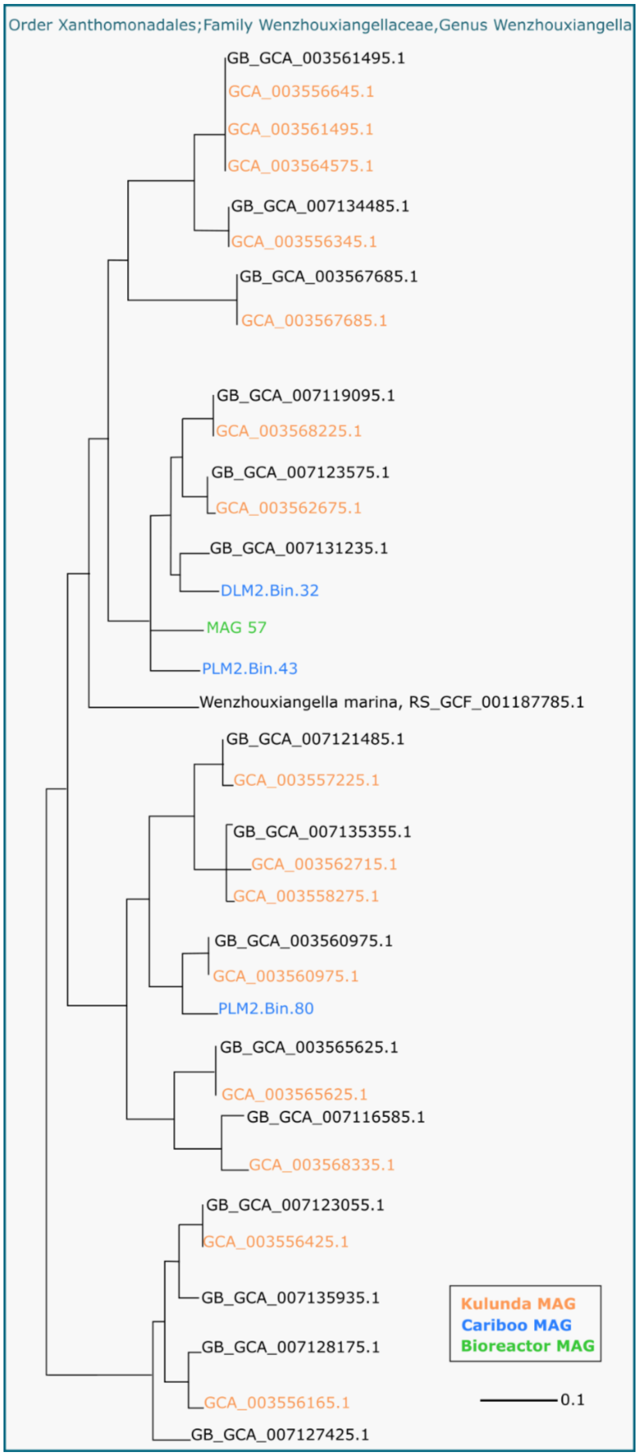

Figure S9 Relationship of the consortium's *Gammaproteobacteria* MAGs to MAGs directly obtained from Cariboo and Kulunda alkaline soda lakes as well as the previously described *Phormidium* OSCR consortium.

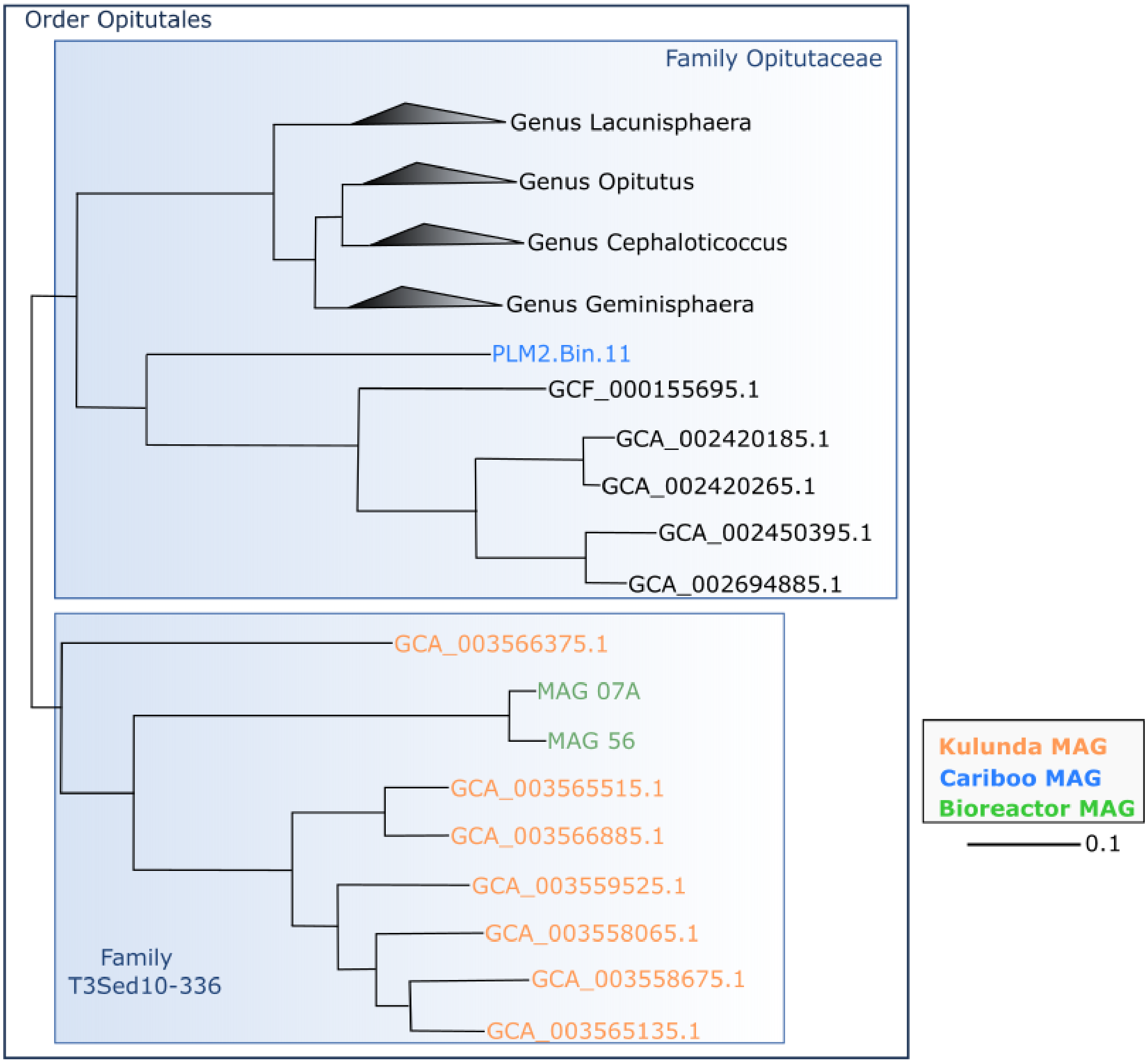

Figure S10 Relationship of the consortium's *Verrucobirobiota* MAGs to MAGs directly obtained from Cariboo and Kulunda alkaline soda lakes as well as the previously described *Phormidium* OSCR consortium.

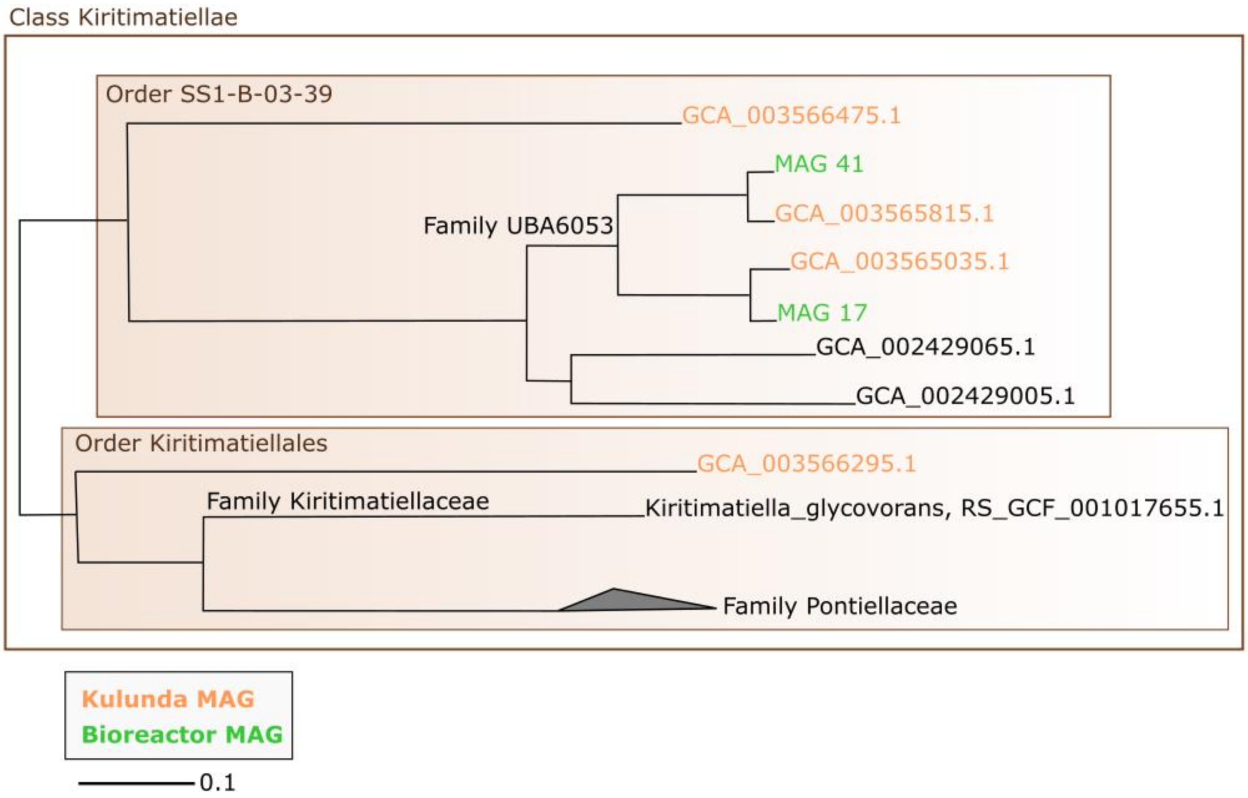

Figure S11 Relationship of the consortium's *Verrucobirobiota* MAGs to MAGs directly obtained from Cariboo and Kulunda alkaline soda lakes as well as the previously described *Phormidium* OSCR consortium.

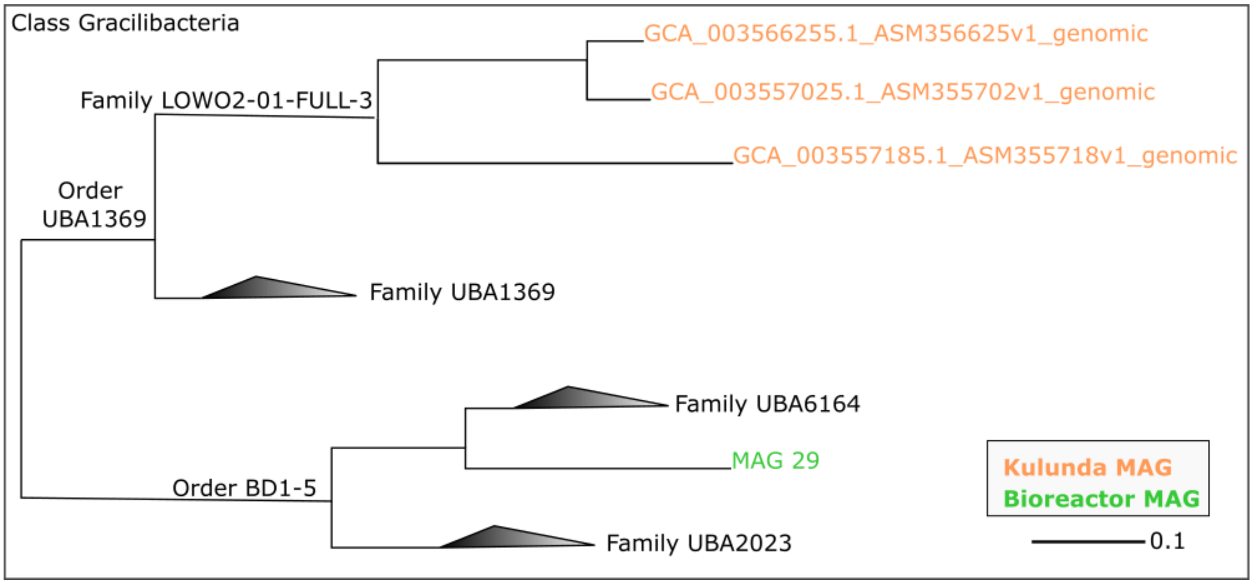

Figure S12 Relationship of the consortium's *Patescibacteria* MAGs to MAGs directly obtained from Cariboo and Kulunda alkaline soda lakes as well as the previously described *Phormidium* OSCR consortium.

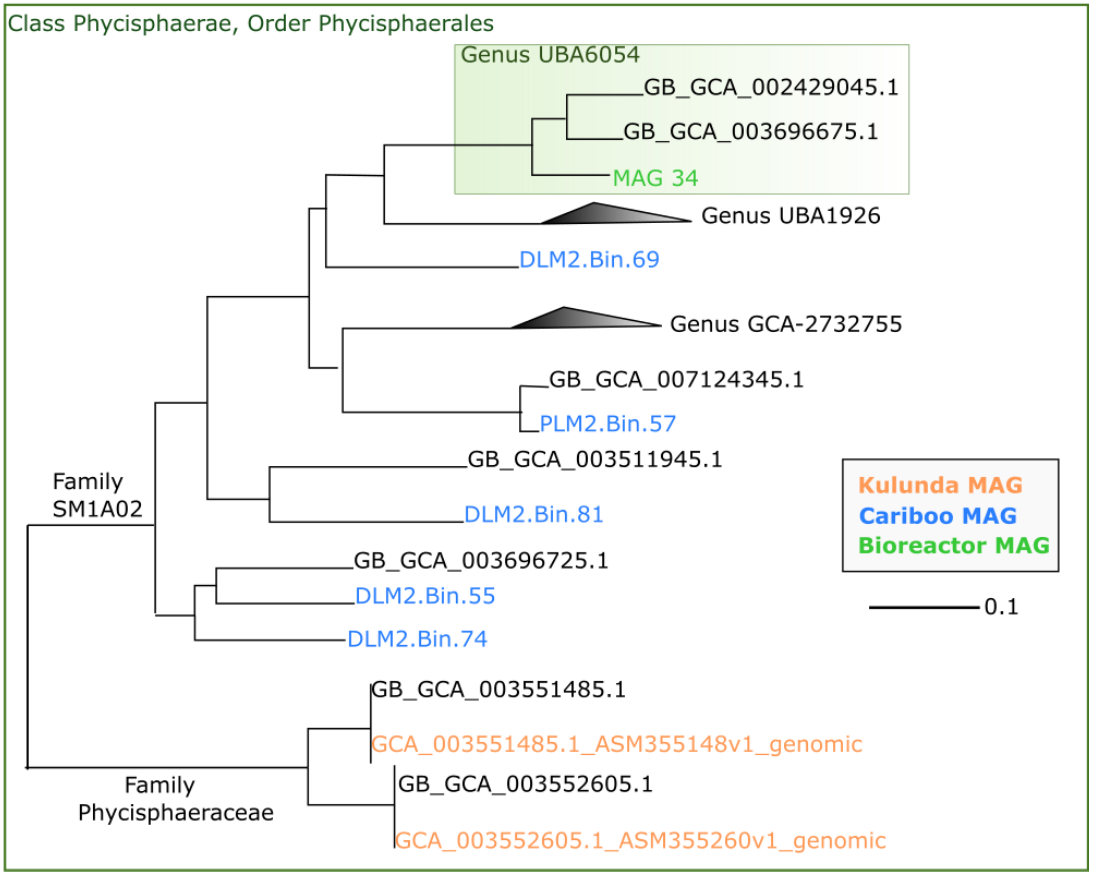

Figure S13 Relationship of the consortium's *Planctomycetota* MAGs to MAGs directly obtained from Cariboo and Kulunda alkaline soda lakes as well as the previously described *Phormidium* OSCR consortium.

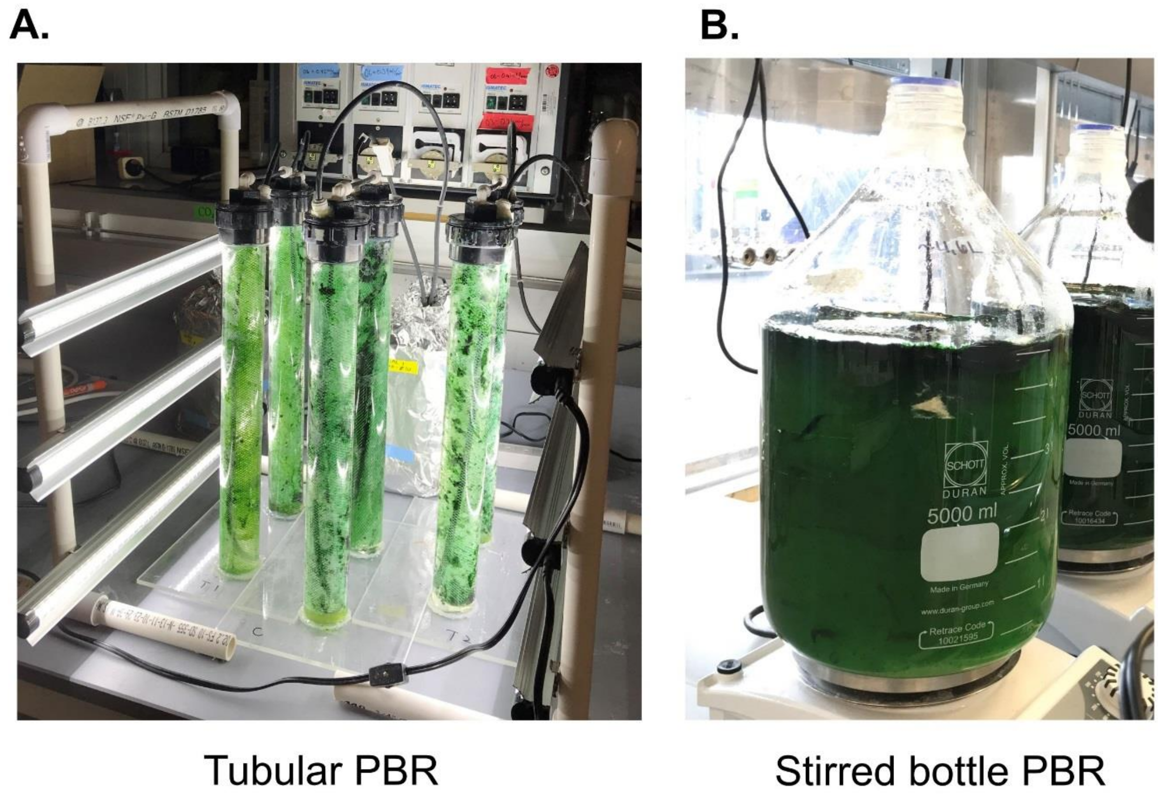

Figure S14 Photographs of the experimental set up of the (A) tubular photobioreactor (B) Stirred bottle photobioreactor
